# Genome-wide sexually antagonistic variants reveal long-standing constraints on sexual dimorphism in fruit flies

**DOI:** 10.1371/journal.pbio.3000244

**Published:** 2019-04-25

**Authors:** Filip Ruzicka, Mark S. Hill, Tanya M. Pennell, Ilona Flis, Fiona C. Ingleby, Richard Mott, Kevin Fowler, Edward H. Morrow, Max Reuter

**Affiliations:** 1 Research Department of Genetics, Evolution and Environment, University College London, London, United Kingdom; 2 Department of Ecology and Evolutionary Biology, The University of Michigan, Ann Arbor, Michigan, United States of America; 3 School of Life Sciences, University of Sussex, Brighton, United Kingdom; 4 College of Life and Environmental Sciences, University of Exeter, Penryn, United Kingdom; 5 The Pirbright Institute, Pirbright, Surrey, United Kingdom; 6 UCL Genetics Institute, University College London, London, United Kingdom; Indiana University, UNITED STATES

## Abstract

The evolution of sexual dimorphism is constrained by a shared genome, leading to ‘sexual antagonism’, in which different alleles at given loci are favoured by selection in males and females. Despite its wide taxonomic incidence, we know little about the identity, genomic location, and evolutionary dynamics of antagonistic genetic variants. To address these deficits, we use sex-specific fitness data from 202 fully sequenced hemiclonal *Drosophila melanogaster* fly lines to perform a genome-wide association study (GWAS) of sexual antagonism. We identify approximately 230 chromosomal clusters of candidate antagonistic single nucleotide polymorphisms (SNPs). In contradiction to classic theory, we find no clear evidence that the X chromosome is a hot spot for sexually antagonistic variation. Characterising antagonistic SNPs functionally, we find a large excess of missense variants but little enrichment in terms of gene function. We also assess the evolutionary persistence of antagonistic variants by examining extant polymorphism in wild *D*. *melanogaster* populations and closely related species. Remarkably, antagonistic variants are associated with multiple signatures of balancing selection across the *D*. *melanogaster* distribution range and in their sister species *D*. *simulans*, indicating widespread and evolutionarily persistent (about 1 million years) genomic constraints on the evolution of sexual dimorphism. Based on our results, we propose that antagonistic variation accumulates because of constraints on the resolution of sexual conflict over protein coding sequences, thus contributing to the long-term maintenance of heritable fitness variation.

## Abstract 2

This study characterises antagonistic loci across the genome of the fruit fly Drosophila melanogaster, finding them to be preferentially associated with variation in coding sequences and to be selectively maintained across worldwide populations of D. melanogaster, and even its sister species D. simulans.

## Introduction

The divergent reproductive roles of males and females favour different phenotypes [1,2]. However, responses to these selective pressures are constrained by a shared genome, leading to ‘sexual antagonism’, in which different alleles at given loci are favoured in the two sexes [1,3–5]. A wealth of quantitative genetic studies has established sexual antagonism as near ubiquitous across a wide range of taxa, including mammals [6] (and humans [7]), birds [8], reptiles [9], insects [10,11], fish [12,13], and plants [14]. Accordingly, sexual antagonism can be considered a major constraint on adaptation [15] and an important mechanism for the maintenance of fitness variation within populations [16].

However, despite its evolutionary importance, we have little understanding of the biological mechanisms underlying this conflict and virtually no empirical data on the identity and evolutionary dynamics of antagonistic alleles [13]. While a small number of individual antagonistic loci have been identified [12,13], these are of limited use for elucidating general properties of loci experiencing sexual antagonism. On a genome-wide scale, previous transcriptomic work in *D*. *melanogaster* has associated antagonistic fitness effects with patterns of gene expression [17]. But despite potentially revealing some of the molecular correlates of fitness variation, this approach cannot distinguish between causal antagonistic loci and their downstream regulatory targets. In humans, genome-wide allele frequency differences between males and females have been used to infer sex-specific selection on viability [18], but this approach neglects important reproductive components of fitness, and furthermore cannot distinguish between loci with opposing fitness effects in each sex (sexually antagonistic loci) and loci where the strength of sexually concordant selection differs between the sexes. It is essential that we characterise causal antagonistic loci underlying lifetime reproductive success in order to understand the adaptive limits to sexual dimorphism and mechanisms of conflict resolution.

To address this shortcoming, we identified sexually antagonistic loci across the *D*. *melanogaster* genome and characterised their functional and evolutionary properties. Specifically, we measured male and female fitness for over 200 hemiclonal lines that had been extracted from LH_M_—the outbred, laboratory-adapted population in which sexually antagonistic fitness effects were first characterised [10,19]. Our fitness measurements estimate lifetime reproductive success in both sexes by replicating the regime under which LH_M_ has been maintained for over 20 years [20]. We combined these fitness data with high-coverage genome sequences [21] and performed a genome-wide association study (GWAS) to map the genetic basis of sexual antagonism. We then examined the properties of candidate antagonistic polymorphisms, including their genomic distribution across the X chromosome and autosomes, their functional characteristics, the genes in which they occur, and their population genomic dynamics across a number of wild populations of *D*. *melanogaster* and two closely related species, *D*. *simulans* and *D*. *yakuba*.

## Results

### Quantitative genetic analyses of sex-specific fitness

We measured the sex-specific fitness of hemiclonal fly lines (*N* = 223) that had been extracted from LH_M_ as part of a previous study [21]. Individuals from each hemiclonal line carry an identical haploid genome comprising all major chromosomes (X, 2, and 3; i.e., about 99% of the total genomic content) paired with a random chromosomal complement from LH_M_ (see Materials and methods and [22,23] for further details). For each line, we measured male fitness as competitive fertilisation success and female fitness as competitive fecundity. The fitness estimates obtained are based on measurements from 25 individual males and females for each hemiclonal line. Assays closely mimic the rearing regime experienced by flies in the base population, thus providing a good proxy for lifetime reproductive success in each sex.

Quantitative genetic analyses confirmed the presence of significant amounts of genetic variation for male and female fitness among the lines assayed. Estimating the genetic variances and covariances between the lines, we found appreciable heritabilities for fitness in both sexes (female $h^{2}\;$= 0.42, 95% CI 0.30–0.54; male $h^{2\;}$ = 0.16, 95% CI 0.04–0.27). Comparable estimates were also obtained by treating single nucleotide polymorphisms (SNPs) as random effects in a linear mixed model and calculating SNP heritability ($h_{SNP}^{2}$) (female $h_{SNP}^{2}$ = 0.59, SD 0.13, *P* < 0.001; male $h_{SNP}^{2}$ = 0.29, SD 0.16, *P* = 0.007). The sex-specific heritabilities estimated via both approaches are consistent with previous estimates in this population [17,24–26]. The intersexual genetic correlation for fitness (r_MF_) did not differ significantly from zero in this sample of genotypes (r_MF_ = 0.15, 95% CI −0.21 to 0.46), which is consistent with some [24,26]—but not all [17,19]—previous work in this population. While antagonism is not dominant in this sample of LH_M_, the absence of a significant positive r_MF_, the high heritability for fitness, and further results (below and S4 Fig) suggest that antagonistic variation is present but overlaid with sexually concordant variation.

**Fig 1:**
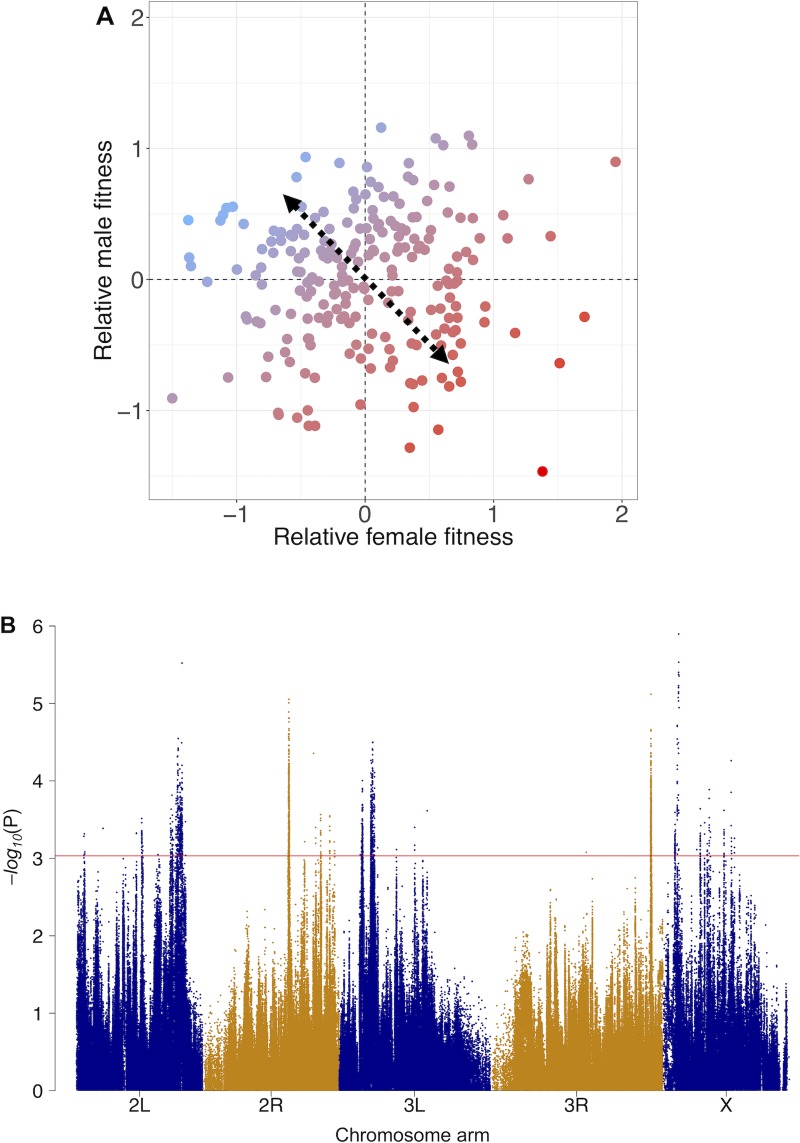
Genome-wide association mapping of sexual antagonism. (A) Relative male and female lifetime reproductive fitness estimates for 223 *D*. *melanogaster* hemiclonal lines. Fitness measures have been normalised, scaled, and centred (see Materials and methods). Colours denote each line’s antagonism index, i.e., their position along a spectrum (dashed arrow) ranging from male-beneficial, female-detrimental fitness effects (blue) to female-beneficial, male-detrimental effects (red). (B) Association of each SNP with the antagonism index along the five major *D*. *melanogaster* chromosome arms, presented as a Manhattan plot in which each point represents the −*log*_*10*_(P) value from a Wald $\chi^{2}\;$association test. Colours denote chromosome arms; the horizontal line represents the Q-value cutoff (0.3) used to define candidate antagonistic SNPs. Data and code underlying this figure can be found at https://doi.org/10.5281/zenodo.2623225. SNP, single nucleotide polymorphism.

We quantified the antagonistic component of fitness variation by calculating an ‘antagonism index’ (Fig 1A). Specifically, we extracted the position of individual fly lines on the axis ranging from extremely male-beneficial, female-detrimental fitness effects to extremely female-beneficial, male-detrimental fitness effects (see Materials and methods). This approach for defining an antagonism index mirrors previous research in this field [27,28] and is analogous to other indices based on combinations of phenotypic measures, such as the widely applied transformation of human height and weight into a body mass index [29]. The antagonism index itself had high SNP heritability ($h_{SNP}^{2}$ = 0.51, SD 0.15, *P* = 0.001), as expected from the heritability of its sex-specific fitness components.

### GWAS of sexual antagonism

To identify putative antagonistic SNPs, we performed a GWAS based on the antagonism index and sequence polymorphism data [21] for 765,764 common (minor allele frequency [MAF] > 0.05) and stringently quality-filtered SNPs across 202 of the 223 lines (see Materials and methods; S1 Fig). We employed a linear mixed model that corrects for between-line relatedness and population structure by incorporating a genetic similarity matrix as a random effect [30] (S2 Fig). Fig 1B presents a Manhattan plot of raw *P* values from SNP-wise association tests along the *D*. *melanogaster* genome. The genomic inflation factor ($\lambda_{median}$ = 0.967) and analyses using alternative, permutation-based significance tests (see Materials and methods) confirmed that the parametric *P* values obtained are robust (S3 Fig).

Because our antagonistic phenotype is a linear combination of male and female fitness, our GWAS could potentially capture variation with sex-limited rather than antagonistic fitness effects. To assess this possibility, we compared GWAS *P* values for the antagonism index with *P* values for a ‘concordant index’, defined as an orthogonal phenotype to the antagonistic index ranging from extremely detrimental fitness effects to extremely beneficial fitness effects in both sexes (S4 Fig). Sex-limited variants should generate symmetrical effects on both the antagonistic and the concordant fitness indices. In contradiction to this expectation, we found that while variation in the antagonism index was dominated by fewer loci with larger effects, variation in the concordant index was distributed across many SNPs with low effect sizes and, consequently, elevated *P* values (S4 Fig). The asymmetry in these patterns indicates that fitness variation in LH_M_ is not due to variants with sex-limited effects. Rather, antagonistic and concordant variation are qualitatively different and appear to be maintained through fundamentally different processes—most likely balancing selection and mutation-selection balance, respectively (see, also, Discussion).

Although no individual antagonistic SNP reached genome-wide significance based on stringent Bonferroni correction (*P* < 6.53 × 10^−8^), our focus was to characterise broad patterns associated with genome-wide antagonistic variation rather than identifying individual antagonistic sites with high confidence. Accordingly, we applied three main approaches to investigate the general properties of antagonistic SNPs and regions. First, we defined 2,372 candidate antagonistic SNPs (approximately 0.3% of all covered SNPs; henceforth ‘antagonistic SNPs’) as SNP positions with false discovery rate (FDR) Q-values < 0.3 (S4 Fig). This threshold achieves a balance between false positives and false negatives that is suitable for our genome-wide analysis and allowed us to contrast the properties of antagonistic and nonantagonistic (Q-value ≥0.3) SNPs. Second, we quantified the importance of different classes of SNPs (defined by chromosomal location or function) by partitioning total SNP heritability of the antagonism index (‘antagonistic $h_{SNP}^{2}$’) into the contribution of each class [31,32]. These contributions can then be tested for deviations from random expectations and interpreted without need for defining significance cutoffs for individual SNPs. Finally, we employed set-based association testing in which the joint effect of a set of SNPs (such as those in a chromosomal window) on the phenotype is assessed. This joint analysis alleviates the multiple testing burden and can be used to define antagonistic windows with more stringent support (Q-value < 0.1). Together, these approaches allowed us to characterise the functional properties and evolutionary dynamics of antagonistic genetic variation.

### Identity and functional properties of antagonistic loci

**Fig 2:**
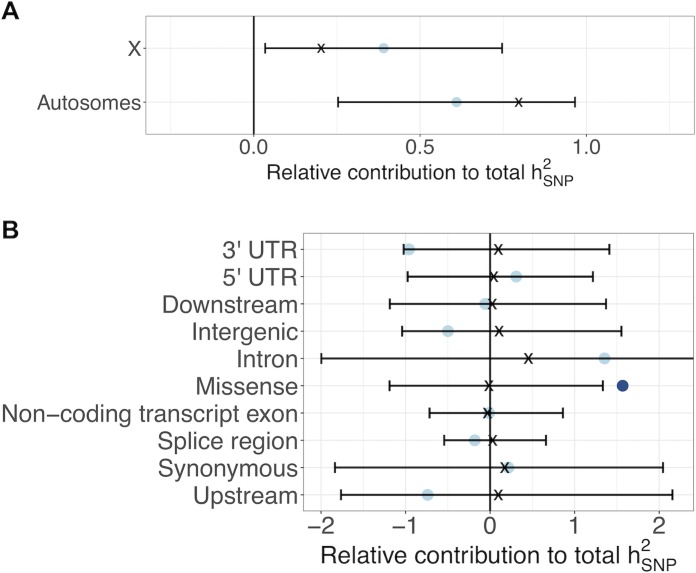
Genomic distribution and functional characteristics of antagonistic variants. (A) Relative contribution of different chromosomal compartments to total SNP heritability of the antagonistic index (i.e., $h_{SNP}^{2}$ estimated for a given compartment divided by total $h_{SNP}^{2}$ across all compartments). Dots represent estimated $h_{SNP}^{2}$ contributions (±95% CI), with expected $h_{SNP}^{2}$ contributions presented as black crosses. (B) Relative contribution of different functional categories to total antagonistic $h_{SNP}^{2}$ (i.e., $h_{SNP}^{2}$ estimated for a given functional category divided by total $h_{SNP}^{2}$ across all categories). Dots represent estimated $h_{SNP}^{2}$ contributions, with expected $h_{SNP}^{2}$ contributions presented as black crosses (±95% CI of the empirical null distribution computed through permutation; see Materials and methods). Colours indicate significant under- or overrepresentation (dark blue: *P* < 0.05; light blue: *P* > 0.05). Data and code underlying this figure can be found at https://doi.org/10.5281/zenodo.2623225. SNP, single nucleotide polymorphism; UTR, untranslated region.

We first examined the genomic distribution of antagonistic variants. The 2,372 antagonistic SNPs were significantly clustered along chromosome arms (median distance: 147 bp on autosomes, 298 bp on the X chromosome; permutation test: *P* < 0.001 for autosomes and X, S5 Fig). Based on observed patterns of linkage disequilibrium (LD) decay in LH_M_ (S1 Fig), we estimated that the antagonistic SNPs form approximately 226 independent clusters. Some previous theory [3,33] and empirical quantitative genetic results [18] suggest that the X chromosome should harbour a disproportionate amount of antagonistic genetic variation because it is hemizygous in males and disproportionately transmitted through females—factors that together favour the invasion and maintenance of X-linked antagonistic polymorphisms in which the male-benefit allele is recessive (but see [34]). This prediction was not borne out by our data. We found that, relative to autosomes, the X chromosome neither contained a disproportionate number of antagonistic SNPs (Z-test, *P* > 0.05, S6 Fig) nor contributed more antagonistic $h_{SNP}^{2}$ than expected (Z-test, *P* = 0.274, Fig 2A).

Our data also provide some of the first insights into the biological functions that underlie sexual antagonism. At the most basic level, our results suggest that antagonism arises primarily because of adaptive conflict over coding sequences. Thus, genomic partitioning revealed that variants that result in missense changes contributed significantly more antagonistic $h_{SNP}^{2}$ than expected from their proportional genomic representation (Fig 2B) and were significantly overrepresented among antagonistic SNPs (S6 Fig). As expected, intergenic regions were underrepresented among antagonistic SNPs and contributed qualitatively less antagonistic $h_{SNP}^{2}$ than expected (Figs 2B and S6). However, we found no evidence that SNP functions involved in expression regulation, such as 3′ untranslated region (UTR), intronic, upstream, or splice region variants, were overrepresented among antagonistic SNPs or $h_{SNP}^{2}$ (Figs 2B and S6).

We next performed a series of analyses to characterise the properties of genes harbouring antagonistic SNPs (one or more antagonistic SNPs within ±5 kb of the gene coordinates). The list of antagonistic genes included some genes known to be involved in sexual differentiation, including *male-specific lethal 1*, *traffic jam*, and *roundabout 2*, the circadian clock gene *period*, and the Golgi-associated transport protein gene *Tango6* that has been previously found to harbour coding sequence polymorphisms shared between *D*. *melanogaster* and *D*. *simulans* [35] (see S1 Table for a complete list of antagonistic genes).

**Fig 3:**
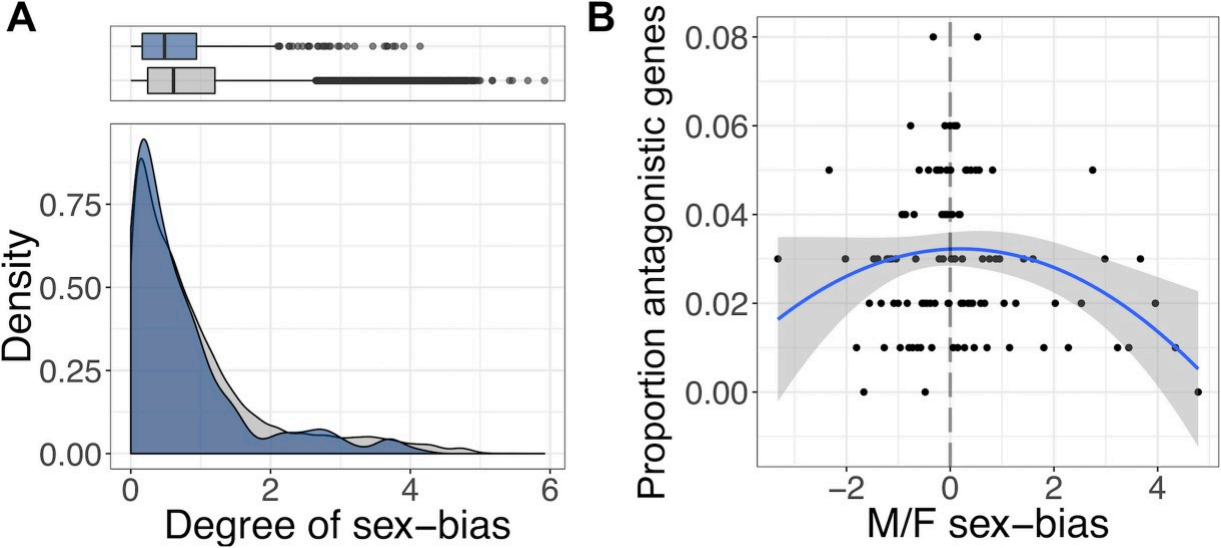
Sex-biased gene expression among antagonistic genes. (A) Distributions of the absolute degree of sex-biased expression for antagonistic (blue) and nonantagonistic (grey) genes. (B) Proportion of genes that are antagonistic across bins of expression sex bias (100 genes per bin). Points represent mean expression level in each bin. Blue curve (±SE) shows the best-fit quadratic model for the relationship between antagonism and expression sex bias. Data and code underlying this figure can be found at https://doi.org/10.5281/zenodo.2623225. F, female; M, male.

We first considered the relationship between antagonism and sex-biased gene expression. This relationship remains poorly delineated, with some studies assuming that sexually antagonistic genes are more likely to be sex biased because sex bias is indicative of the action of antagonistic selection [36]. Others, in contrast, infer that less sex-biased genes are more likely to be antagonistic because they have higher intersexual genetic correlations, and polymorphisms will result in more opposed fitness effects under sexually antagonistic selection [37]. Using estimates of sex bias in gene expression extracted from the Sebida database [38], we could address this question directly. Doing so, we found that antagonistic genes had lower sex bias in gene expression than nonantagonistic genes. This pattern was detectable on several levels. In qualitative terms, fewer antagonistic genes than expected by chance were classified as showing significant sex-biased gene expression (observed = 188, expected = 212, 11.3% deficit, $\chi_{1}^{2}$ = 7.78, *P* = 0.005). In quantitative terms, antagonistic genes had a lower degree of absolute sex bias than did nonantagonistic genes (W = 1,309,700, *P* < 0.001, Fig 3A) and the probability of genes being antagonistic peaked at zero sex bias (generalised linear model [GLM] with quadratic term, $\chi_{1}^{2}$ = 6.20, *P* = 0.013, Fig 3B). We also tested the predictions of a recent ‘Twin Peaks’ model [39], which proposes that antagonistic genes should be enriched among genes with intermediate sex bias in expression and depauperate among genes with low and high sex bias. Yet, we did not detect any enrichment for antagonistic genes among intermediately sex-biased genes as predicted by this model (comparison of quadratic versus fourth-degree polynomial GLM: $\chi_{2}^{2}$ = 0.67, *P* = 0.714).

Gene Ontology (GO) analysis revealed little evidence for preferential association of antagonistic variation with specific biological processes. Only one term, ‘sodium-channel-regulator-activity’, was significant after correction for multiple testing (Q-value = 0.013). However, this annotation is shared by only a few genes (*N* = 5), a cluster of four of which carry antagonistic SNPs. It thus appears that antagonism is not enriched in genes involved in specific functions. We also did not find a significant overlap between the antagonistic genes identified here and genes that had previously been shown to have sexually antagonistic expression patterns (opposing relationships between expression level and fitness in males and females [17]; observed overlap = 41, expected overlap = 36, $\chi_{1}^{2}$ = 0.59, *P* = 0.44). This discrepancy is not necessarily unexpected. Leaving aside the fact that the previous study of antagonistic expression was based on a small sample of genotypes [40] and did not correct for their kinship, large overlap need not exist between loci that show antagonistic expression patterns and those that harbour causal antagonistic sequence variants. For instance, antagonistic expression patterns in a large number of genes can be caused by a very small number of genetic changes in regulatory genes. Equally, the numerous coding sequence variants that we detect can cause antagonism in the absence of expression differences.

Finally, we tested whether antagonistic variation is enriched in genes that are likely to be subject to pleiotropic constraints. Elevated gene pleiotropy—defined here as ‘molecular gene pleiotropy’ [41], in which a gene performs several functions and affects several traits—has been proposed to make the evolution of sex-specific expression more difficult, because altered expression could alleviate antagonism in some traits but have a deleterious effect on other traits mediated by a gene. These pleiotropic effects could then impede the resolution of sexual antagonism via the evolution of sex-biased expression [42]. We did not find support for this hypothesis in our data, as there was no association between antagonism and higher levels of pleiotropy, measured either as tissue breadth τ [43] (W = 1,264,700, *P* = 0.70) or as the number of protein–protein interactions (PPIs) [44] (F_1,5276_ = 2.43, *P* = 0.12). This implies that pleio-tropy—at least as captured by τ and PPIs—does not contribute significantly to maintaining sexually antagonistic genetic variation.

### Comparative population genomic analyses of balancing selection

In addition to assessing the functional properties of antagonistic loci, we also investigated the population genetic effects of sexual antagonism. Population genetic models predict that the opposing sex-specific fitness effects of antagonistic alleles generate balancing selection [16,45–47], resulting in elevated levels of genetic polymorphism at antagonistic loci. Having identified candidate antagonistic variants, we can test this prediction by comparing levels of polymorphism at antagonistic and nonantagonistic loci. Examining levels of polymorphism in LH_M_, we found that antagonistic sites have higher MAFs than nonantagonistic sites (W = 1,024,100,000, *P* < 0.001) and that regional polymorphism (Tajima’s D, measured within 1,000-bp windows along the chromosome arms) is higher at antagonistic windows (those with Q-value <0.1 in a window-based GWAS) than nonantagonistic windows (Q-value ≥0.1; F_1,195208_ = 279.6, *P* < 0.001).

However, although these patterns are suggestive, looking at polymorphism within LH_M_ is potentially problematic because variation in MAF will generate ascertainment bias: the power to detect antagonistic effects is higher at more polymorphic sites, and candidates will therefore tend to show above-average polymorphism, even in the absence of significant balancing selection. A more robust approach is to use data from independent populations and ask whether polymorphism in those populations is greater at antagonistic than at nonantagonistic sites, while controlling for a range of potential confounders. Specifically, we compared levels of polymorphism at antagonistic and nonantagonistic sites while controlling for (i) MAF ascertainment bias (i.e., increased GWAS power to detect antagonistic effects at more polymorphic sites in LH_M_), (ii) differences in genome-wide estimates of linked selection, which could inflate polymorphism near antagonistic sites if they are situated in regions of the genome less affected by selective sweeps or background selection, and (iii) pseudo-replication due to close-by sites in LD showing correlated signals of both antagonistic fitness effects and polymorphism.

**Fig 4:**
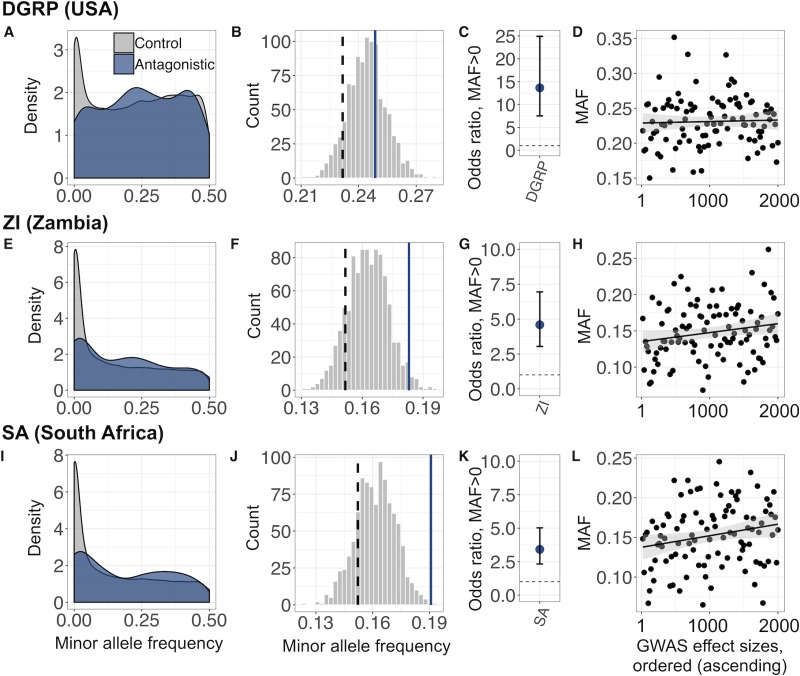
SNP-based signatures of balancing selection associated with antagonistic variants in three independent populations (DGRP, ZI, and SA). (A,E,I) Spectra of raw MAF for LD-independent antagonistic (blue) and nonantagonistic (‘Control’, grey) SNPs. (B,F,J) Distribution of mean MAFs for 1,000 sets of LD-independent nonantagonistic SNPs that have been frequency matched and linked-selection matched to LH_M_ antagonistic SNPs (‘Analysis A’; see Materials and methods). Blue line denotes mean MAF of antagonistic SNPs; black dashed line denotes mean MAF of nonantagonistic SNPs without matching for LH_M_ MAF or linked selection. (C,G,K) Odds ratio (±95% CI) that a site is polymorphic (i.e., has the same alleles as in LH_M_ and has MAF >0) as a function of its absolute GWAS effect size (regression coefficient), while controlling for LH_M_ MAF and genome-wide linked selection (‘Analysis B’; see Materials and methods). An odds ratio >1 (dashed horizontal line) indicates that sites with higher absolute effect sizes are more likely to be polymorphic in a given population. Of the LD-independent sites considered, the number and percentage of sites with MAF > 0 were *N* = 31,092 (91.3%), *N* = 25,578 (77.3%), and *N* = 21,659 (68.7%) in the DGRP, ZI, and SA populations, respectively. (D,H,L) Mean MAF across 100 sets of LD-independent SNPs, presented in ascending order by absolute GWAS effect size (‘Analysis C’; see Materials and methods). Each set of LD-independent SNPs has been matched for LH_M_ MAF and genome-wide estimates of linked selection. For visualisation purposes, a linear regression line (±95% CI) is shown. Data and code underlying this figure can be found at https://doi.org/10.5281/zenodo.2623225. DGRP, *Drosophila* Genetic Reference Panel; GWAS, genome-wide association study; LD, linkage disequilibrium; MAF, minor allele frequency; SA, South Africa; SNP, single nucleotide polymorphism; ZI, Zambia.

**Fig 5:**
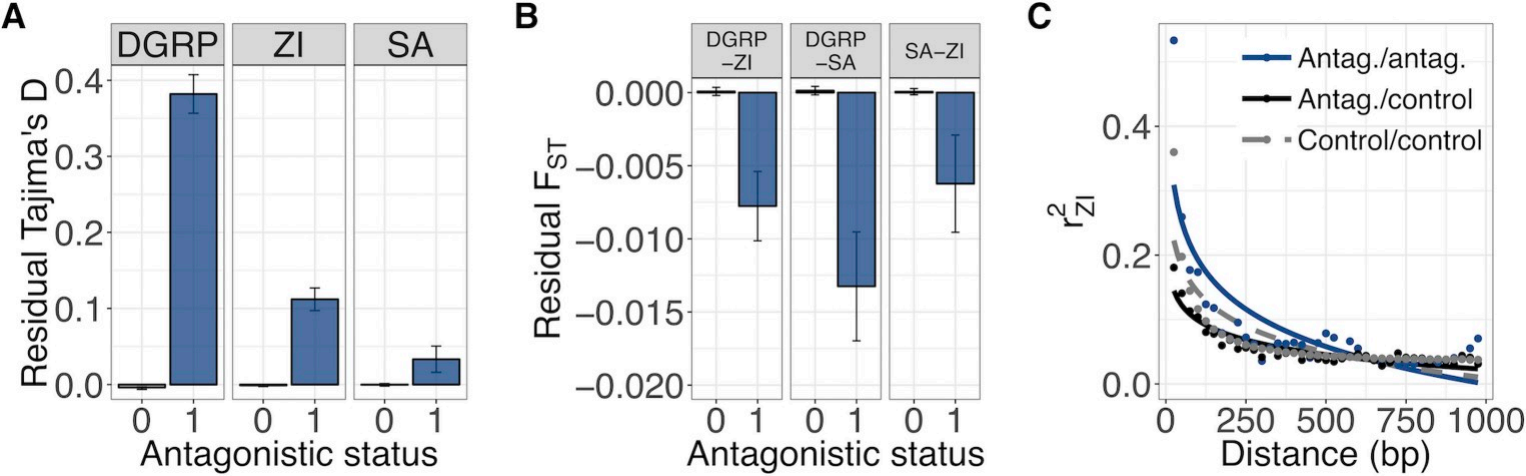
Regional and LD-based signatures of balancing selection associated with antagonistic variants in three independent populations (DGRP, ZI, and SA). (A) Mean (±SE) residual Tajima’s D (i.e., the residuals of a regression of Tajima’s D on genome-wide estimates of linked selection) for antagonistic windows (blue; ‘antagonistic status = 1’) and nonantagonistic windows (grey; ‘antagonistic status = 0’). (B) Mean (±SE) residual F_ST_ (i.e., the residuals of a regression of F_ST_ on genome-wide estimates of linked selection) for antagonistic and nonantagonistic windows. Because these are residuals of a regression, residual F_ST_ does not vary between 0 and 1. (C) LD (r^2^) in the ZI population between pairs of antagonistic SNPs (blue, ‘Antag./antag.’), pairs of nonantagonistic SNPs (grey, ‘Control/control’), and mixed pairs (black, ‘Antag./control’). Points represent mean r^2^ across 25-bp bins; r^2^ is modelled as a declining exponential function of distance (fitted lines). Data and code underlying this figure can be found at https://doi.org/10.5281/zenodo.2623225. Antag., antagonistic; DGRP, *Drosophila* Genetic Reference Panel; LD, linkage disequilibrium; SA, South Africa; SNP, single nucleotide polymorphism; ZI, Zambia.

We performed analyses controlling for these effects (see Materials and methods for details), first using publicly available polymorphism data from the *Drosophila* Genetic Reference Panel [48,49] (DGRP), a collection of 205 wild-derived inbred lines. Like LH_M_, the DGRP was established from a North American *D*. *melanogaster* population. Given the relatively recent colonisation of the continent by *D*. *melanogaster* (about 150 years [50]), the two populations are closely related. We found that antagonistic SNPs had elevated MAFs in the DGRP. Yet, owing to the close relationship between LH_M_ and the DGRP (and the resulting similarity in allele frequencies), this difference was not statistically significant (empirical *P* = 0.322, Fig 4A and 4B; ρ= 0.010, *P* = 0.66, Fig 4D). Nevertheless, we also found that the probability of SNPs being polymorphic (i.e., to have MAF > 0) in the DGRP increased with absolute GWAS effect size ($\chi_{2}^{2}$ = 76.23, *P* < 0.001, Fig 4C). This shows that—once the level of polymorphism in LH_M_, linked selection, and pseudo-replication are accounted for—LH_M_ SNPs are more likely to also be polymorphic in the DGRP if they are more closely associated with antagonism. This evidence for antagonism-driven balancing selection at individual SNPs was corroborated by patterns of regional polymorphism. Thus, Tajima’s D was significantly higher in 1,000-bp antagonistic windows than in nonantagonistic windows (F_1,115477_ = 224.6, *P* < 0.001, Fig 5A). Overall, these analyses show that the heritable phenotypic variation in sex-specific fitness that can be generated and maintained by sexual antagonism is mirrored by a signal of increased polymorphism at the underlying genetic loci.

A key, yet so far unresolved, question is whether antagonistic polymorphisms are mainly short-lived and population specific or persist over prolonged periods of time. The analyses of polymorphism in the DGRP shed some light on this question, demonstrating that antagonistic polymorphisms are maintained at least over the tens to hundreds of years (hundreds to a few thousand generations) that separate this population from LH_M_. In order to assess signals of balancing selection over longer time spans, we repeated these analyses with polymorphism data from two populations in *D*. *melanogaster*'s ancestral sub-Saharan distribution range, in Zambia (ZI, 197 genomes from phase 3 of the *Drosophila* Population Genomics Project [51]) and South Africa (SA, 118 genomes from South Africa [52]). Just as in the DGRP, we found that antagonism generated a clear signature of balancing selection in these ancestral population samples. Analyses based on binary categories showed that antagonistic SNPs had significant excess MAF in ZI and SA compared with nonantagonistic SNPs (ZI: empirical *P* = 0.024, Fig 4E and 4F; SA: empirical *P* = 0.001, Fig 4I and 4J; see also S7 Fig), while analyses based on GWAS effect sizes showed that sites with stronger evidence for antagonistic effects were again more likely to be polymorphic (ZI: $\chi_{2}^{2}$ = 53.03, *P* < 0.001, Fig 4G; SA: $\chi_{2}^{2}$ = 39.41, *P* < 0.001, Fig 4K) and also have more elevated MAFs (ZI: ρ = 0.047, *P* = 0.037, Fig 4H; SA: ρ = 0.055, *P* = 0.014; Fig 4L; see also S7 Fig). At a larger chromosomal scale, antagonistic windows had significantly higher polymorphism (Tajima’s D) than nonantagonistic windows (ZI: F_1,116099_ = 60.63, *P* < 0.001; SA: F_1,110954_ = 4.24, *P* = 0.039; Fig 5A). Furthermore, they also exhibited lower population differentiation between all pairs of populations (measured as F_ST_; DGRP-ZI: W = 50,667,000, *P* < 0.001; DGRP-SA: W = 50,975,000, *P* < 0.001; SA-ZI: W = 55,322,000, *P* < 0.001; Fig 5B), in line with balancing selection maintaining similar frequencies across distant populations.

In addition to elevated polymorphism in antagonistic regions of the genome, we also found evidence for increased LD—another hallmark of balancing selection [53,54]. We compared local LD (<1,000 bp, measured as r^2^) between pairs of antagonistic sites, pairs of nonantagonistic sites, and ‘mixed’ site pairs (consisting of an antagonistic and a nonantagonistic SNP) in the ZI population, which is most phylogenetically distant from LH_M_ and where a signal of LD should be weakest in the absence of long-term balancing selection. Consistent with selection, we found that pairs of antagonistic sites had higher LD in this population than pairs of nonantagonistic sites (W = 8,346,500,000, *P* < 0.001, Fig 5C). They also had higher LD relative to mixed pairs (W = 33,823,000, *P* < 0.001, Fig 5C). Thus, high LD between antagonistic sites is not an artefact of unusually low levels of recombination near antagonistic regions, but instead reflects the action of long-term balancing selection.

The signal of conserved maintenance of antagonistic polymorphisms across populations of *D*. *melanogaster* raises the possibility that sexual antagonism is maintained over yet longer timescales. To assess this, we asked whether antagonistic loci are more likely to be detected as polymorphic than nonantagonistic loci in two closely related species, *D*. *simulans* and *D*. *yakuba*, that are separated from *D*. *melanogaster* by about 1 and 3 million years, respectively [55]. An enrichment of antagonistic loci among such ‘*trans*-specific’ polymorphisms—while controlling for possible confounders, as previously—would indicate that antagonistic selection maintains allelic variants across species boundaries.

**Fig 6:**
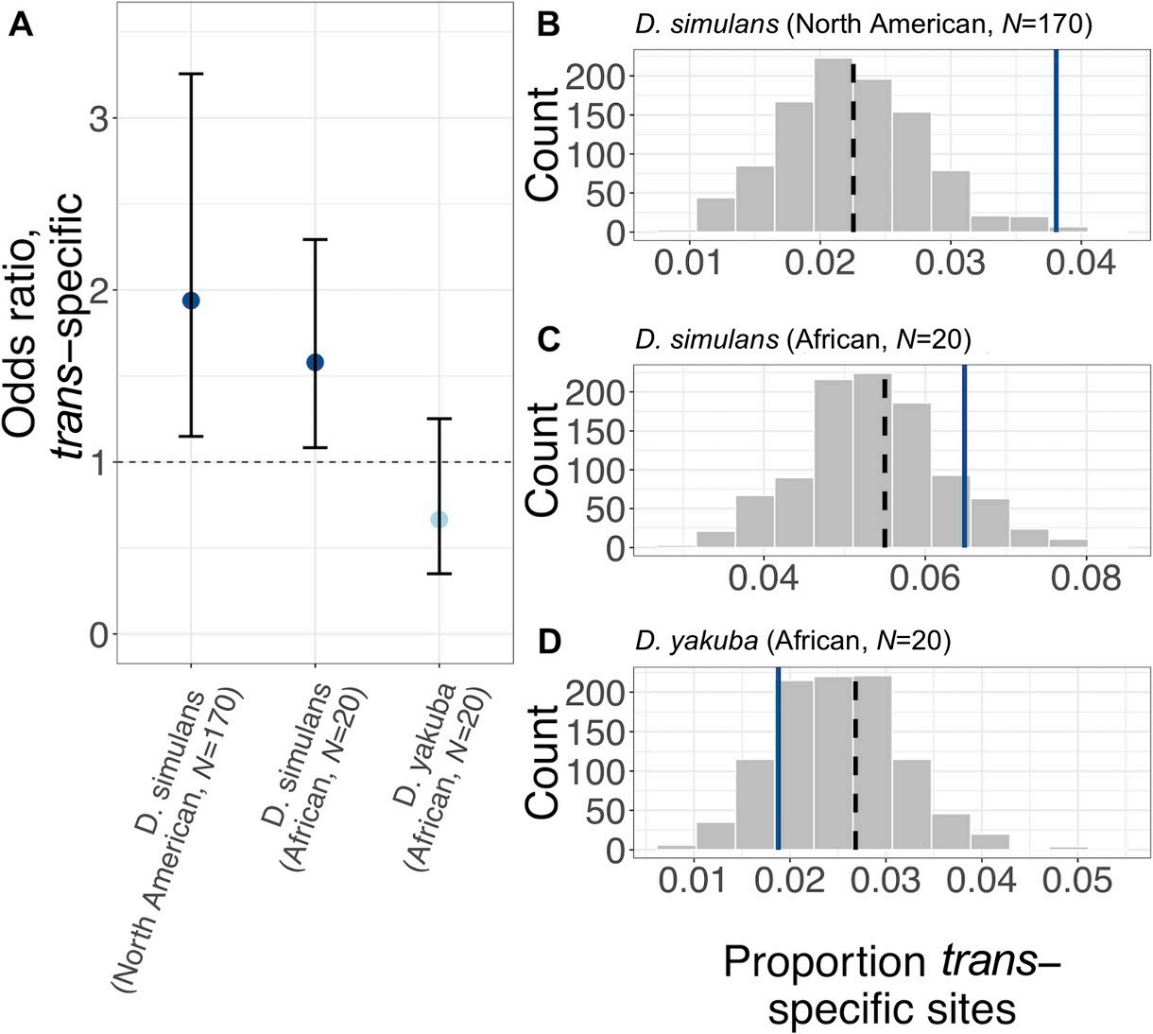
Signatures of balancing selection associated with antagonistic variants in two sister species, *D*. *simulans* and *D*. *yakuba*. (A) Odds ratio (±95% CI) that a polymorphism’s *trans*-specific status varies with absolute GWAS effect size (regression coefficient), controlling for LH_M_ MAF and genome-wide linked selection (see Materials and methods). An odds ratio >1 indicates that sites with higher effect sizes are more likely to be *trans*-specific. The relationship between *trans*-specific status and effect size is presented for three datasets: a panel of 170 North American *D*. *simulans* genomes (left), 20 African *D*. *simulans* genomes (middle), and 20 African *D*. *yakuba* genomes (right). Colours indicate significant under- or overrepresentation (dark blue: *P* < 0.05; light blue: *P* > 0.05). Of the LD-independent sites considered in each sample, the number and percentage of *trans*-specific sites were *N* = 3,608 (2.2%), *N* = 7,466 (5.5%) in the 170- and 20-genome *D*. *simulans* datasets, respectively, and *N* = 2,760 (2.7%) in the *D*. *yakuba* dataset. (B) Histogram of the proportion of *trans*-specific polymorphisms for 1,000 sets of LD-pruned nonantagonistic SNPs that have been frequency and linked-selection matched to antagonistic SNPs. Blue line denotes mean proportion of *trans*-specific antagonistic SNPs; black dashed line denotes mean proportion of *trans*-specific nonantagonistic SNPs without any correction for LH_M_ MAF or linked selection. *Trans*-specific status was determined by considering polymorphism data from a panel of 170 North American *D*. *simulans* genomes. (C) Same as B, with *trans*-specific status derived from polymorphism data from a panel of 20 African *D*. *simulans* genomes. (D) Same as B., with *trans*-specific status derived from polymorphism data from a panel of 20 African *D*. *yakuba* genomes. Data and code underlying this figure can be found at https://doi.org/10.5281/zenodo.2623225. GWAS, genome-wide association study; LD, linkage disequilibrium; MAF, minor allele frequency; SNP, single nucleotide polymorphism.

Consistent with this hypothesis, we found that antagonistic loci are enriched among *trans*-specific polymorphisms in *D*. *simulans*. Thus, we detected a significant positive correlation between absolute GWAS effect size in LH_M_ and the probability that a polymorphism is *trans*-specific among a panel of 170 North American *D*. *simulans* genomes [56] ($\chi_{2}^{2}$ = 6.13, *P* = 0.013, Fig 6A) and a panel of 20 sub-Saharan African *D*. *simulans* genomes [57] ($\chi_{2}^{2}$ = 5.65, *P* = 0.017, Fig 6A). A similar pattern was detectable when comparing antagonistic/nonantagonistic sites as a binary classification among the dataset of 170 North American genomes (empirical *P* = 0.001, Fig 6B). In the smaller panel of 20 African genomes, antagonistic loci also displayed elevated polymorphism, but the excess was not statistically significant (empirical *P* = 0.100, Fig 6C). We finally tested whether antagonistic loci are enriched among *trans*-specific polymorphisms in *D*. *yakuba*, using polymorphism data from a panel of 20 African genomes [57]. In this species, we could not detect an enrichment of antagonistic loci, whether using GWAS effect sizes as a continuous predictor of *trans*-specific status ($\chi_{2}^{2}$ = 1.59, *P* = 0.207, Fig 6A) or comparing antagonistic/nonantagonistic SNP classes (empirical *P* = 0.844, Fig 6D).

Taken together, these comparative population genomic analyses demonstrate that the antagonistic allelic variation identified in LH_M_ is neither recent nor population specific. To a significant degree, balancing selection maintains antagonistic variation over timescales that extend beyond the extension of the species range out of Africa, more than 10,000 years ago [50], and across species boundaries to *D*. *simulans*. The elevation in polymorphism generated by antagonism is not, however, indefinite, as indicated by the absence of a signal in the relatively more distant *D*. *yakuba*.

## Discussion

Our study provides the first, to our knowledge, analysis of the identity, function, and evolution of genome-wide sexually antagonistic sequence polymorphisms. Remarkably, we find that genetic variation at antagonistic loci is stably maintained across *D*. *melanogaster* populations throughout the species’ distribution range, and across species boundaries into *D*. *simulans*. These results demonstrate that the targets of antagonistic selection have been largely conserved for many millennia [50,58–60]—and hundreds of thousands of generations—and that a number of antagonistic polymorphisms have arisen and persisted since the speciation event between *D*. *melanogaster* and *D*. *simulans*, approximately 1 million years ago. It is possible that our GWAS only captures a subset of antagonistic variants, i.e., those that remain polymorphic in the constant laboratory environment to which the LH_M_ population has adapted. Nevertheless, the geographical stability and low turnover in antagonistic sequence variation that we detect among these loci imply that a significant proportion of the adaptive conflict between males and females is rooted in a fundamental aspect of the biology of the sexes. As a consequence, it persists even in the face of environmental variation [61] and is relatively unaffected by the adaptation of populations to the range of environmental conditions that they encountered during their colonisation of the globe [50,59,62], or the continuous adaptive evolution that occurs within temperate populations over the course of the seasons [63].

While sexual antagonism can generate balancing selection, the range of parameters over which simple models of antagonistic selection predict this to be the case is restrictive [16]. The fact that antagonistic alleles have persisted over such long timescales therefore suggests that additional forces operate to stabilise polymorphism. One prime candidate for such a force is dominance reversal, in which, at a polymorphic site, the allele with the beneficial effect is dominant in each sex. Such sex-specific dominance drastically widens the range of male and female fitness effects over which antagonistic selection actively maintains polymorphism, improving the prospects for the long-term maintenance of antagonistic allelic variation [34,64]. An empirical example of dominance reversal at a putative sexually antagonistic polymorphism has recently been documented in salmon [13], and a quantitative genetic study in seed beetles inferred a large and significant contribution of sex-specific dominance to sexually antagonistic fitness variation [28]. Based on this evidence, it is plausible to assume that dominance reversal will also be involved in the long-term maintenance of antagonistic polymorphisms in fruit flies.

The long-term stability of sexually antagonistic polymorphisms further suggests that the evolutionary constraints on sexual dimorphism inherent in antagonism are difficult to resolve. While we do not find any evidence that there is elevated pleiotropy among genes experiencing ongoing conflict, the persistence of antagonism fits with our finding that antagonistic polymorphisms are highly enriched for missense variants. While antagonistic selection on expression levels can be accommodated by the gradual evolution of sex-specific gene expression [65], adaptive conflicts over coding sequences can only be resolved through a complex multistep process [66] of gene duplication, sex-specific subfunctionalisation of coding sequences, and the evolution of differential expression of the two paralogues [67] (there is some evidence for this process in *D*. *melanogaster*, in which paralogues have been shown to acquire male-biased expression, but there is little correlation between sex-biased paralogue expression and sequence divergence [68]). The requirement for gene duplication, in particular, would be expected to constitute a severely limiting barrier for this route towards resolution, as suitable mutation events will be exceedingly rare. This large barrier to resolution, and the resulting stochasticity in which antagonisms will undergo resolution, may also help to explain the lack of GO enrichment observed among antagonistic genes.

The fact that antagonistic polymorphisms are enriched in coding regions and among genes with lower than average levels of expression sex bias may seem at odds with some recent work, which has placed emphasis on conflicts over gene expression. For instance, sex-biased gene expression has been shown to correlate positively with genome-wide polymorphism in flycatchers [36], while male-biased genes showed lower polymorphism in guppies [69], and alleles under sex-specific viability selection have been shown to exhibit intermediate levels of sex-biased expression in humans [39]. However, the conclusions from these studies are not straightforward to interpret because antagonistic selection is not measured directly; instead, it is inferred from expression bias or polymorphism, and these inferred effects are potentially confounded by partially sex-limited or relaxed selection. Furthermore, the results we have produced here do not imply that antagonism over gene expression levels is absent (indeed, we find a number of highly sex-biased antagonistic genes). Nevertheless, they do suggest that coding regions play a disproportionate role in ongoing and long-term constraints on adaptive evolution between the sexes—a feature that may extend beyond sexually antagonistic polymorphisms and affect other types of trade-offs. Interestingly, this is mirrored in polymorphisms associated with fluctuating selection in *Drosophila* [63] and with trade-offs between traits in humans [70], both of which are enriched among variants with missense effects. Conversely, genetic loci underlying local adaptation are found in excess among gene regulatory regions relative to coding regions [71], implying that regulatory regions can respond to context-specific selection faster. Thus, our results corroborate an emerging pattern whereby regulatory regions facilitate adaptation while protein-coding regions constrain it, at least over more modest evolutionary time spans.

We find no convincing evidence that the X chromosome is enriched for antagonistic variation, in contradiction to classic theory [3]. This discrepancy could be due to the presence of sex-specific dominance, as mentioned previously, and which is predicted to shift enrichment of antagonism from the X to the autosomes [34]. Antagonistic loci could also interact epistatically—a common process [72] that similarly favours the accumulation of autosomal antagonism [73]. However, while these general effects might explain the lack of X enrichment, our result also contradicts previous empirical findings obtained in the LH_M_ population itself [18], which found that the X chromosome contributed disproportionally to antagonistic fitness variation. The previous study was based on a much smaller sample of genomes, with large uncertainties about the estimated chromosomal contributions. It was also performed more than 10 years ago and much closer to the establishment of LH_M_ as a laboratory population. Accordingly, the discrepancy to our results might in part be explained by stronger genetic drift on the X chromosome relative to autosomes, which could in turn lead to a disproportionate loss of X-linked antagonistic polymorphisms [45]. Finally, given that our GWAS focuses on common variants, the fitness effects of low-frequency X-linked antagonistic variants might not be adequately captured, potentially exacerbating the absence of an enrichment of antagonistic variation on the X.

In contrast to some previous studies [19,25,74] (but see [24,26]), we do not detect a significant negative r_MF_ among this sample of genotypes. One potential explanation for this result could be that current fitness variation in LH_M_ is caused by variants with sex-limited effects. However, this hypothesis is difficult to reconcile with the contrasting properties of variants mapping to the antagonism and concordant fitness indices. Our data instead suggest that while some antagonistic variation may have been lost through genetic drift [45,47,75], the remainder is overlaid with highly polygenic sexually concordant variation [76]. This explanation fits with the observation that most traits in *D*. *melanogaster* exhibit strong positive intersexual genetic correlations [37,77,78]. This provides a large target for generating sexually concordant deleterious variants that would subsequently be maintained at mutation-selection balance—with fitness effects that have been documented in numerous experimental studies (see [79] for review). A further contribution to the nonnegative r_MF_ that we observed could come from sexually concordant variants evolving under balancing selection due to their antagonistic pleiotropic effects on unmeasured components of fitness (e.g., juvenile fitness). Finally, we acknowledge that the hemiclonal fitness design does not allow us to capture the totality of fitness variation in LH_M_. For example, the fact that we measure fitness in a heterozygous background implies that unconditionally deleterious recessive alleles segregating at low frequencies are not adequately screened; additionally, small differences between the assay environment and the rearing regime typically experienced by LH_M_ flies—despite all attempts at minimising such differences—may have contributed some measurement error in our fitness estimates.

In addition to addressing long-standing gaps in our understanding of sexual antagonism, this study also informs broader debates regarding the forces that maintain genetic variation for fitness in natural populations, which remain insufficiently understood [80]. By identifying genome-wide antagonistic variants and linking these loci to signatures of balancing selection in independent populations, our results supplement a growing body of evidence suggesting that balancing selection can influence patterns of genetic variation on a genome-wide scale [81], rather than a very limited number of isolated loci, as is sometimes assumed [82]. For example, previous quantitative genetic analyses have shown that levels of genetic variation observed among populations of *Drosophila* [79] and other species [83] far exceed the levels predicted under mutation-selection balance alone, implying an important role for balanced polymorphisms. Moreover, a recent genomic study in *D*. *melanogaster* has linked candidate loci under opposing selection between seasons with long-term elevations in genome-wide polymorphism [63], emphasising the importance of fluctuating balancing selection across the genome. Sexually antagonistic selection should contribute particularly strongly to the buildup of balanced polymorphisms, given that there is abundant evidence for sex-specific selection in nature [4,84] and that many traits exhibit strong positive genetic correlations between the sexes [37,77]. Together, these factors generate permissive conditions for the evolution of sexually antagonistic polymorphisms relative to alternative sources of balancing selection [46,85].

Our study provides a starting point from which to clarify the relative role of antagonistic and nonantagonistic modes of balancing selection towards the maintenance of genetic variation. But it also provides the foundation for further work on the genetics of sexual antagonism, including elucidating its functional basis and testing theories regarding its resolution (e.g., [86]). Together, these future studies will allow us to better understand how males and females can, or cannot, respond to sex-specific selection and adapt to their respective reproductive roles.

## Materials and methods

### LH_M_ hemiclones

LH_M_ is a laboratory-adapted population of *D*. *melanogaster* that has been maintained under a highly controlled rearing regime since 1996 [23]. A random sample of 223 genetic lines was created from the population [21] using a hemiclonal approach [22]. Individuals of each line carry an identical haploid genome comprising the major chromosomes X, 2, and 3. Crosses with flies from custom stocks allow the generation of many replicate individuals—males and females—that carry a line’s X-2-3 haplotype alongside a random chromosomal complement from the LH_M_ population that can be assayed for fitness. In our experiment, 16 randomly sampled LH_M_ females were used to supply the chromosomal complements for each line, sex, and block.

### Fitness measurements

Lifetime adult reproductive fitness of males and females of each line was measured using proven assays designed to mimic the LH_M_ rearing regime [87]. For male fitness, we measured competitive fertilisation success by setting up competition vials containing 5 hemiclonal males from a given line, 10 competitor *bw* males, and 15 virgin *bw* females. After two days, *bw* females were isolated into individual vials containing no additional yeast and left to oviposit for 18 hours. On day 12 post egg laying, progeny were scored for eye colour. Male fitness was calculated as the proportion of offspring sired by the 5 hemiclonal males (those with wild-type eye colour), combining progeny data from the 15 oviposition vials. This assay was repeated five times in a blocked design; estimates for each line were therefore based on fitness measurements from 25 hemiclonal males. As expected for flies carrying a homozygous phenotypic marker mutation, *bw* males are slightly inferior to wild-type males (they sired approximately 46.4% instead of the expected two thirds of offspring across all fitness assays). However, they provide a meaningful competitive standard for our male fitness measures, as evidenced by the significant heritability in male mating success.

Female fitness was measured as competitive fecundity. Competition vials containing 5 virgin hemiclonal females from a given line, 10 competitor *bw* females, and 15 *bw* males were set up. Two days later, the 5 hemiclonal females were isolated into individual vials and left to oviposit for 18 hours. These vials were immediately chilled at 4°C and fecundity was measured by counting the number of eggs laid per female. This assay was replicated five times in a blocked design; each line estimate therefore measured the fitness of 25 hemiclonal females.

Fitness data were subjected to quality control and preprocessing in preparation for quantitative genetic and association analysis. Male fitness data from competition vials in which not all 5 focal males were present at the end of the assay were removed from further analysis. Similarly, we omitted female oviposition vials in which fewer than 2 eggs were present (indicating partial sterility or failure to mate) or in which the female had died over the course of the assay. For each sex, fitness measurements were then first box-cox transformed to be normally distributed within each block, then scaled and centred. To calculate SNP heritabilities and for association analysis, data from each block were averaged to obtain one fitness estimate for each line and sex.

### Quality control of whole-genome sequences

We used previously published whole-genome sequences generated from the hemiclonal lines analysed here [21] (available at https://zenodo.org/record/159472). Details about DNA extraction, library preparation, sequencing, read processing, and SNP calling are provided in the original publication. Prior to the association analysis performed here, further site-level quality filtering steps were performed in vcftools [88] and PLINK [89]. First, individual variant calls based on depth <10 and genotype quality <30 were removed. Second, individuals with >15% missing positions were removed. Third, positions with poor genotype information (<95% call rate) across all retained individuals were discarded. Finally, given the relatively small sample size of the dataset as a whole and the low power of an association test for rare variants, we retained only common variants (MAF > 0.05) for further analysis. From an initial dataset of 220 hemiclones containing 1,312,336 SNPs, this yielded a quality-filtered dataset of 765,980 SNPs from 203 hemiclones.

To detect outliers, we examined LH_M_’s population structure using principal components analysis (PCA). Overlapping SNP positions from the 203 LH_M_ genomes and from an outgroup population (DGRP [48]) consisting of 205 whole-genome sequenced individuals were used as input to construct a genetic similarity matrix. This set of SNPs was pruned for LD such that no two SNPs with r^2^ > 0.2 within 10 kb remained. The leading PC axes were extracted in LDAK (‘Linkage Disequilibrium Adjusted Kinships’) [90]. After removal of one outlier (see S1 Fig), the final dataset used for association analysis contained 202 individuals and 765,764 SNPs.

### Heritability analyses

We estimated the variance-covariance matrix for fitness and sex-specific residual variances by fitting a model using MCMCglmm [91] implemented in R. Specifically, we fitted the model $Y_{ijk}\;=\;X_{ij}+\varepsilon_{ijk}$, where $Y_{ijk}$ is the scaled and centred fitness of individual *k* of genotype *j* and sex *i*, $X_{ij}$ is the sex-specific random effect of genotype *j* in sex *i*, and $\varepsilon_{ijk}$ describes the sex-, genotype-, and individual-specific residual. The genotypic fitness effects in males and females follow a bivariate normal distribution $X_{ij}\sim N(0,G)$, where

$$G=\left(\begin{array}{cc} \sigma_{G,m}^{2} & Cov_{G,mf}\\ Cov_{G,mf} & \sigma_{G,f}^{2} \end{array}\right)$$

is the genetic variance-covariance matrix across sexes (composed of male and female additive genetic variances $\sigma_{G,m}^{2}$ and $\sigma_{G,f}^{2}$ and the intersexual genetic covariance ${Cov}_{G,mf}$). Residuals follow a normal distribution $\varepsilon_{ijk}\sim N(0,\sigma_{R,i}^{2})$, where $\sigma_{R,i}^{2}$ is the sex-specific residual variance, and are assumed to be uncorrelated across sexes.

From these variance estimates, we calculated male and female heritabilities of fitness as $h_{i}^{2}=2\sigma_{G,i}^{2}/\left(\sigma_{G,i}^{2}+\sigma_{R,i}^{2}\right)$, where the subscript *i* indicates either male or female. The factor 2 in the heritability calculation reflects the fact that with the hemiclonal approach, individuals assayed share half their genetic material (the hemizygous hemiclonal genome). The intersexual genetic correlation was calculated as $r_{mf}=Cov_{G,mf}/\left(\sqrt{\sigma_{G,m}^{2}}\sqrt{\sigma_{G,f}^{2}}\right)$. The quantitative genetic parameters $h_{m}^{2},\;h_{f}^{2}$, and $r_{mf}$ were calculated for each sample from the Monte Carlo Markov chain. From these series of values, we obtained point estimates (averages) and 95% credible intervals (using the function HPDintervals).

As a complementary approach, we estimated the SNP heritability ($h_{SNP}^{2}$) of male and female fitness in LDAK [90]. This approach uses restricted maximum likelihood (REML) [92] to fit a linear mixed model that expresses the vector of phenotypes *Y* as a function of genome-wide SNP genotypes, treated as random effects:

$$Y\sim N\left(0,\sigma_{SNP}^{2}K+\;\sigma_{e}^{2}I\right)$$

where **K** is the kinship matrix, $\sigma_{SNP}^{2}$ a vector of additive genetic variances for each SNP, $\sigma_{e}^{2}$ the vector of residual variances, and **I** an individual identity matrix. SNP heritability is then estimated as $h_{SNP}^{2}=\sigma_{SNP}^{2}/\left(\sigma_{SNP}^{2}+\sigma_{e}^{2}\right)$.

LDAK corrects for local linkage when calculating SNP heritabilities to avoid inflation of $h_{SNP}^{2}$ in clusters of linked sites that otherwise arises because several SNPs tag the same causal polymorphism. SNPs are weighted inversely proportional to their local linkage, such that SNPs in high LD contribute less to $h_{SNP}^{2}$ than SNPs in low LD. This model has been shown to substantially improve heritability estimates across a wide range of traits [31]. LDAK also allows us to set the parameter α that determines how SNPs are weighted by their MAF (as ${MAF}^{\alpha }$) when calculating the kinship matrix **K**. We used the default of α = −0.25, which provides a steeper relationship between MAF and $h_{SNP}^{2}$ than the value of −1 that is frequently used in studies on humans. Significance of $h_{SNP}^{2}$ estimates was assessed by permuting phenotype labels 1,000 times, recalculating $h_{SNP}^{2}$ on each permutation, as above, and calculating the number of permuted estimates that exceeded the observed.

### Quantification and association analysis of sexual antagonism

To identify loci underlying sexual antagonism, we followed the approach employed by Berger and colleagues [27] and subsequent researchers [28] and defined an ‘antagonism index’ (Fig 1A). Specifically, we rotated the coordinate system of the male and female fitness plane by 45 degrees, by multiplying the matrix of fitness coordinates (average male and female fitness estimate for each hemiclonal line) by a rotation matrix:

$$R=\left[\begin{array}{cc} -1/\sqrt{2} & -1/\sqrt{2}\\ -1/\sqrt{2} & 1/\sqrt{2} \end{array}\right]$$

This transforms the matrix of hemiclonal male and female fitness values into positions on a bivariate coordinate system (of dimension 2 × 202 lines) with one sexually antagonistic and one sexually concordant axis. These positions represent the values of the ‘antagonism index’ used to map antagonistic genetic variation, as well as the ‘concordant index’ used for comparative purposes (see section ‘Comparison of antagonistic and concordant variants’ below).

The univariate approach we employ for measuring antagonistic effects has several benefits. First, it mirrors the approach taken by previous researchers in this field [27,28]. Second, univariate approaches have proved effective in other contexts (e.g., the study of human obesity [29]). Third, although additional power to disentangle sex-limited from antagonistic effects could be gained by employing bivariate analysis, this method would additionally require combining significance measures on two axes to distinguish variants with significant sexually antagonistic effects from those with sex-limited effects. Our approach, in contrast, provides a simple and clear measure of antagonistic effects that is appropriate for our main focus, which is to draw general conclusions about the properties of antagonistic variants.

We calculated the SNP heritability of the antagonism index (‘antagonistic $h_{SNP}^{2}$’) in LDAK, following the same procedure and settings as those for estimating sex-specific SNP heritabilities.

We performed a GWAS by applying a linear mixed model to test the effect of allelic variants at each SNP on the antagonism index, while including the kinship matrix as a random effect to account for the heritable portion of genetic variation attributable to kinship between individuals. This approach has been shown to effectively control the false positive rate and increase power to detect true associations in samples with moderate degrees of population structure and close relatedness, such as LH_M_ [30,93]. The GWAS was implemented in LDAK (settings as above) and a Wald $\chi^{2}$ test was used to generate *P* values for each position (Fig 1B).

To estimate the extent to which genetic confounding affects GWAS *P* values, we performed two procedures. First, we calculated a genomic inflation factor [94] (median $\chi_{obs}^{2}$/median $\chi_{exp}^{2}$ using the GenABEL [95] package) (S2 Fig). Second, we used a permutation-based test to compute empirical *P* values and compared these *P* values with those computed through a Wald $\chi^{2}$ test (S3 Fig). In order to perform permutation testing when individuals have different degrees of genetic resemblance, we used the method in Nicod and colleagues [96]. In brief, we modelled the phenotypic variance-covariance matrix as $V\;=\;K\sigma_{g}^{2}+I\sigma_{e}^{2}$, where K is the genetic similarity matrix, I is the identity matrix, and $\sigma_{g}^{2},\sigma_{e}^{2}$ are the genetic and environmental variance components. This is the standard mixed-model decomposition. We then computed the matrix square root $V\;=\;A^{2}\;$and multiplied the phenotypes yand genotypes G by the matrix $A^{-1}\;$to generate a transformed dataset $A^{-1}y,\;A^{-1}G$ whose variance matrix is the identity I. Thus, the transformed phenotypes ${z\;=\;A}^{-1}y$ are all equally related, and so are exchangeable for permutations. We then performed 100,000 permutations of *z* and performed the mixed-model GWAS (which, in the case of the transformed data, becomes an ordinary least squares model) to determine the empirical *P* value of each SNP. Empirical and parametric *P* values were highly correlated (S3 Fig).

### Defining candidate antagonistic SNPs and regions

We corrected for multiple testing using an FDR approach and converted *P* values into Q-values using the method of Benjamini and Hochberg [97]. We defined antagonistic SNPs as sites with FDR Q-values <0.3 and nonantagonistic SNPs as sites with Q-values ≥0.3. Given the observed distribution of Q-values (see S4 Fig), choosing a cutoff of 0.3 allowed us to achieve a suitable balance between false positives and false negatives. These candidate antagonistic SNPs show a near-perfect overlap with the set of SNPs exhibiting the lowest empirical *P* values (as obtained through the permutation-based approach), further supporting their robustness as candidates (S3 Fig).

For analyses that consider larger genomic regions (windows), we ran a set-based association test implemented in LDAK (options using ‘–calc-genes-reml’, ‘ignore-weights YES’, and α = −0.25). The test calculates set-wide $h_{SNP}^{2}$ via REML, corrects for local relatedness using the predictors in each window, and computes a *P* value using a likelihood ratio test (LRT). The sets we used were 1,000-bp windows (500-bp step), defined according to *Drosophila* Reference 5 genome coordinates and subsequently converted (using the liftOver tool [98]) to Release 6 coordinates. This was a necessary step, as publicly available polymorphism data were mapped to Release 5 of the *D*. *melanogaster* genome, whereas the GWAS data were mapped to Release 6. We then calculated window-based Q-values from the LRT *P* values and defined antagonistic windows as those with a Q-value <0.1.

### Comparison of antagonistic and concordant variants

To support our inference that the GWAS of the antagonism index captures genetic variation with antagonistic effects, we compared these GWAS *P* values with *P* values estimated from a GWAS of the ‘concordant index’ (S4 Fig), which describes the position of individual fly lines on the axis ranging from extremely male- and female-detrimental fitness effects to extremely male- and female-beneficial fitness effects. We also transformed these *P* values into Q-values (as described above) and compared Q-values for both indices. We did not analyse sexually concordant variants further, owing to the absence of any sites that are significantly associated with this phenotype (minimum Q-value = 0.78).

### Genomic distribution of antagonistic SNPs

To estimate the number of independent antagonistic regions, we performed LD clumping in PLINK [89]. We used a significance threshold of 0.00093 for the index SNP (the maximum, least significant *P* value across all antagonistic SNPs) and clustered (‘clumped’) neighbouring antagonistic SNPs by specifying an r^2^ threshold of 0.4 and a distance threshold of 10 kb.

We also quantified the clustering by calculating the median distance between all pairs of adjacent antagonistic SNPs across chromosome arms (S5 Fig). We did this separately for the autosomes and X chromosome, to accommodate for the lower SNP density on the X chromosome. We tested for significant clustering by using a permutation test, in which antagonistic/nonantagonistic labels were permuted among all SNPs, distances between adjacent SNPs labelled as ‘antagonistic’ after permutation were recalculated as before, and the median distance recorded. This process was repeated 1,000 times in order to generate a null distribution of median distances. The significance of clustering among true antagonistic SNPs was calculated as the proportion of median distances in the null distribution that were lower than or equal to the true median distance.

To examine the proportional contribution of autosomal and X-linked antagonistic variants to total $h_{SNP}^{2}$, we used two complementary methods. First, we partitioned the genome into X chromosome and autosome subsets, and calculated $h_{SNP}^{2}$ via REML in LDAK, each subset in turn (settings as above) (Fig 2A). The observed proportion of $h_{SNP}^{2}$ contributed by each compartment was then compared with the expected proportion (i.e., the proportion of LD-weighted predictors belonging to each compartment). We tested whether the two compartments contributed significantly more $h_{SNP}^{2}$ than expected using a two-sample Z-test. Second, we compared the proportion of antagonistic SNPs (Q-value < 0.3) with the proportion of all SNPs mapping to each chromosomal compartment, using Z-tests (S6 Fig). The under- or overrepresentation of antagonistic SNPs (deficit or excess of antagonistic compared with all SNPs) in each compartment is therefore unaffected by differences in SNP density between chromosome arms, such as the lower density on the X chromosome.

### Functional analyses of antagonistic loci

We used the variant effect predictor (Ensembl VEP [99]) to map SNPs to functional categories. We partitioned total antagonistic $h_{SNP}^{2}$ into functional subsets, and estimated the observed proportion of $h_{SNP}^{2}$ contributed by each subset using REML in LDAK (settings as above) (Fig 2B). We then used a permutation test to compare observed and expected $h_{SNP}^{2}$ for each functional category, in which we shifted genome-wide annotations to a random starting point along a ‘circular genome’. This procedure breaks the relationship between each SNP and its annotation while preserving the order of annotations and their associated LD structure [100]. $h_{SNP}^{2}$ was recalculated via REML for each of 1,000 permuted datasets, and two-tailed *P* values were determined as the sums of permuted estimates with more extreme absolute values than the observed. As a complementary approach, we compared the proportion of antagonistic SNPs with the proportion of all SNPs mapping to each functional category (S6 Fig). We then assessed enrichment for each functional category in turn using Z-tests.

We also used the VEP to map SNPs to genes. We included extended gene regions (± 5 kb of gene coordinates, VEP default) in our gene definition. To gain preliminary insights into the functions of antagonistic genes we used the Gorilla [101] GO tool, with FDR correction for multiple testing across GO terms. All genes covered in the final SNP dataset were used as the background set.

To examine the relationship between antagonistic genes and sex-biased gene expression, we used the Sebida online database [38] to annotate genes as having either sex-biased or unbiased expression profiles (meta-class identifier). We then used a $\chi^{2}$ test to compare the sex-biased expression status of antagonistic and nonantagonistic genes. We additionally examined the quantitative degree of sex bias using this same dataset (Fig 3A). We took the absolute value of the log2-transformed ‘M_F’ bias variable, such that large values indicate more extreme sex bias in expression, irrespective of whether this bias is towards males or females. We compared the distributions of this variable between antagonistic and nonantagonistic genes using a Wilcoxon rank-sum test. Finally, to examine the shape of the relationship between antagonism and expression sex bias, we modelled the binary candidate status of genes (antagonistic/nonantagonistic) as a function of expression sex bias using GLMs with binomial error structure (logit link function). We tested for a quadratic relationship between sex bias and candidate status (Fig 3B) by comparing a second-degree polynomial model with a model including only the linear effect of sex bias and its square. We also assessed the fit of a fourth-degree polynomial model by comparing it with the second-degree model. Model comparisons were performed using LRTs based on the $\chi^{2}$ distribution.

To assess the degree of overlap between antagonistic genes identified here and those associated with sexually antagonistic expression patterns in a previous study [17], we included only genes covered in both datasets, and only those genes in both datasets that were adult expressed. To determine whether genes were adult expressed, we used the *Drosophila* gene expression atlas (FlyAtlas [102]). Conservatively, we considered a gene ‘adult-expressed’ if its transcript was detected as present in at least one library of one adult-derived sample. We then used a $\chi^{2}$ test to assess the degree of overlap between the datasets.

We used the tissue-specificity index (τ) to compare pleiotropy between antagonistic and nonantagonistic genes. We used gene expression data from FlyAtlas [102] to get average expression values for each gene and in each tissue and then calculated τ as

$$\tau =\frac{\sum_{i=1}^{n}\left(1-\hat{x_{i}}\right)}{n-1}$$

where $\hat{x_{i}}\;=\;x_{i}/\underset{1\leq i\leq n}{\text{max}}\left(x_{i}\right)$ is the proportional expression level of the gene in tissue i, and n is the number of tissues. We then excluded sex-limited genes from this dataset by removing those genes that fell into the most extreme 5% quantiles for the female- and male-biased M/F ratio distributions from the Sebida data. Finally, we compared values of τ for antagonistic and nonantagonistic genes using a Wilcoxon rank-sum test.

As an additional proxy for pleiotropy, we examined the number of PPIs between antagonistic and nonantagonistic genes. We used the physical interactions table from FlyBase [103] to summarise the total number of PPIs for all genes and then compared antagonistic and nonantagonistic genes using a GLM with quasipoisson error structure to account for overdispersion.

### Comparative population genomic data

To analyse SNP polymorphism outside the LH_M_ population, we used publicly available population genomic data from three wild *D*. *melanogaster* populations. The first is an introduced population from North America (DGRP [48,49]: 205 whole-genome sequences derived from inbred lines). The two others come from *D*. *melanogaster*'s ancestral distribution range in sub-Saharan Africa (ZI: 197 whole-genome sequences derived from haploid embryos; SA: 118 whole-genome sequences derived from inbred lines, which combine data from subpopulations 'SD' and 'SP' and have very low population differentiation [52]).

All genome sequences were downloaded as FASTA files from the Drosophila Genome Nexus website (www.johnpool.net/genomes.html). These files had been generated following standardised alignment and quality-filtering steps [51] and were further quality filtered for admixture and identity by descent using scripts provided on the Genome Nexus website. We used SNP-sites [104] to call SNPs and convert the multiple sequence alignments to VCF format. Allele frequencies in the three populations were calculated using vcftools [88]. We further excluded tri-allelic and poorly covered sites (call rate <20).

### SNP-based analyses of balancing selection

To test whether antagonistic sites are associated with signatures of balancing selection at the level of individual SNPs, we used three comparison populations (DGRP, ZI, SA) and only considered data for those positions that appeared as SNPs in the LH_M_. Our analyses focussed on SNP polymorphism (MAF) in relation to antagonistic fitness effects but controlled for a number of potential confounders (the exact details are elaborated on in the following paragraphs). First, they controlled for MAF ascertainment bias, which could be caused by the higher MAF of antagonistic relative to nonantagonistic sites in LH_M_ itself. Second, they controlled for ‘linked selection’, which could differentially affect antagonistic/nonantagonistic sites if each class of site is nonrandomly distributed across the genome. Third, they controlled for pseudo-replication between neighbouring SNPs due to LD. Finally, all SNP-based analyses focussed on within-population comparisons, so demographic differences between populations did not confound our analyses.

We performed three main analyses (A, B, and C). In analysis A, we asked whether antagonistic SNPs showed greater polymorphism than nonantagonistic SNPs and compared MAF (corrected for confounding effects) at the two classes of sites. In analysis B, we assessed the sharing of SNP polymorphism between LH_M_ and a comparison population in relation to antagonistic fitness effects by looking for an association between absolute GWAS effect size and the probability that an SNP shows polymorphism (i.e., has MAF > 0 for the same variants as in LH_M_) in each non-LH_M_ population. In analysis C, we assessed the relationship between polymorphism and antagonistic fitness effects in a quantitative way and looked for an association between absolute GWAS effect size and MAF in each population.

In our first analysis comparing antagonistic/nonantagonistic MAF (analysis A; Fig 4B, 4F and 4J), we first LD pruned the LH_M_ dataset by clumping (in PLINK) to avoid pseudo-replication due to correlations between SNPs. For antagonistic sites, we used the 226 index SNPs identified in the previous clumping (see section ‘Genomic distribution of antagonistic SNPs’). For nonantagonistic sites, a nonantagonistic SNP was randomly chosen as an index SNP and clumped by clustering all SNPs within 10 kb with r^2^ > 0.4. Pruning in this manner reduced the original dataset of 765,764 SNPs to 36,316 ‘LD-independent’ SNPs. Note that LD typically decays within 1 kb in wild *D*. *melanogaster* populations and that the degree of LD pruning used in the above analyses is therefore highly stringent (analyses that use less stringent thresholds return similar results).

We then used this LD-independent dataset to compare MAF between antagonistic and nonantagonistic SNPs (we assigned MAF = 0 to sites that were monomorphic in a comparison population and those in which a comparison population was polymorphic for variants other than those segregating at that site in the LH_M_). We did this using a Monte Carlo approach in which, 1,000 times, we drew 226 nonantagonistic ‘control’ SNPs and carefully matched them to the 226 antagonistic SNPs in terms of their LH_M_ MAF and genome-wide estimates of ‘linked selection’ [105] (estimates of linked selection quantify local recombination rates and proximity to functional sequences in *D*. *melanogaster* and thereby account for factors that affect polymorphism along the genome, such as background selection and selective sweeps). The matching procedure first corrected LH_M_ MAF for linked selection by taking the residuals of a linear regression of LH_M_ MAF on estimates of linked selection. Then, sets of 226 nonantagonistic SNPs were drawn to match the linked selection-corrected LH_M_ MAF distribution of the 226 antagonistic SNPs, and for each set we calculated the mean MAF in the comparison population. The 1,000 sets generated in this way provided a null distribution of MAFs for nonantagonistic sites in each comparison population. *P* values for deviations in polymorphism between antagonistic and nonantagonistic sites were then calculated by comparing, in each population, the mean MAF of the 226 antagonistic SNPs with the null MAF distribution.

In our second analysis (analysis B; Fig 4C, 4G and 4K), we used the same LD-pruned dataset but considered SNP polymorphism as a function the whole spectrum of effect sizes, rather than a binary split of SNPs into antagonistic/nonantagonistic categories. We performed a logistic regression, modelling the binary response of whether or not an LH_M_ SNP is polymorphic in a given population (i.e., has MAF > 0 and harbours the same alleles as those found in LH_M_) as a function of the absolute effect size of the SNP in LH_M_, while including LH_M_ MAF and estimates of linked selection as covariates. To assess significance, we performed a $\chi^{2}$ test comparing a model that did include absolute effect size to one that did not.

Our third analysis (analysis C; Fig 4D, 4H and 4L) was similar to analysis B, but rather than asking whether the presence of polymorphism in another population varied with antagonistic effect size in LH_M_, it assessed whether MAF in another population varied with effect size. To do so, we used the same LD-pruned dataset and performed binning in two dimensions, by residual LH_M_ MAF (20 quantiles) and GWAS effect size (100 quantiles). We then drew one SNP from each of these MAF/effect size bins (2,000 SNPs in total), recorded the MAF for each in the comparison population of interest, and finally correlated these MAF values with effect sizes using a Spearman’s rank correlation. Under the hypothesis of antagonism-mediated balancing selection, we would expect to see a positive correlation between MAF and effect sizes across these matched sets of SNPs, with SNPs with higher effect sizes tending to be associated with higher MAFs in a comparison population under consideration than SNPs with lower effect sizes.

To provide insights into the functions of long-term balanced antagonistic sites, we repeated the above analyses for each functional category in turn in the ZI population (S7 Fig). Because of the small number of antagonistic SNPs in each category and the low resulting power of this analysis, we did not perform LD clumping prior to binary antagonistic/nonantagonistic comparisons. In effect size–based analyses, in which the pool of possible sites to draw from was larger, LD clumping was performed.

### Window-based analyses of balancing selection

We performed genome-wide sliding window analyses (1,000-bp windows, 500-bp step size) to investigate regional signatures of balancing selection (Fig 5A). Tajima's D, which compares SNP polymorphism (nucleotide diversity, π) with SNP abundance (the Watterson estimator, $\theta_{W}$), was compared for windows defined as antagonistic (Q-value < 0.1) or nonantagonistic (Q-value ≥ 0.1) from the set-based analysis (see section ‘Defining candidate antagonistic SNPs and regions’). Under the hypothesis that antagonism generates balancing selection, Tajima’s D is expected to be elevated in antagonistic windows. We calculated Tajima’s D for each comparison population using PopGenome [106] in R. As in SNP-based analyses, we incorporated estimates of linked selection [105] (estimated in 1,000-bp windows) by calculating the residuals of a regression of Tajima’s D on estimates of linked selection. Because estimates of linked selection were not available for windows on the X chromosome, we instead used estimates of the recombination rate on this chromosome [107]. We then used a GLM, assuming Gaussian error structure, to compare residual Tajima’s D between antagonistic and nonantagonistic windows.

We also tested for another signature of balancing selection, reduced population differentiation (Fig 5B). Measures such as F_ST_ are often considered problematic because they do not correct for the dependency of F_ST_ on local levels of polymorphism [108]. However, the availability of genome-wide estimates of linked selection in *D*. *melanogaster* allowed us to incorporate this confounding variable explicitly. We therefore estimated F_ST_ over windows, using PopGenome, correcting F_ST_ for linked selection in a way analogous to that used for Tajima’s D. Because the distribution of F_ST_ values is not normally distributed, we contrasted residual F_ST_ between antagonistic and nonantagonistic windows using Wilcoxon rank-sum tests.

### Linkage-based analyses of balancing selection

We examined the extent to which antagonistic haplotypes are selectively maintained by investigating whether antagonistic SNPs have unusually high LD in the ZI population, the population that is most distant from LH_M_ and in which levels of LD between antagonistic SNPs should be weakest in the absence of long-term balancing selection. Thus, for all SNPs situated within 1,000 bp of one another in ZI and that were also covered in LH_M_ (i.e., SNPs that could be inferred to be either antagonistic or nonantagonistic), we calculated pairwise LD (r^2^) in PLINK. We then compared r^2^ values between pairs of antagonistic SNPs and two control pairs: nonantagonistic pairs and ‘mixed’ pairs (antagonistic/nonantagonistic) (Fig 5C). Comparing pairs of antagonistic SNPs to the mixed pairs allowed us to consider only SNPs located close to an antagonistic SNP, thus effectively controlling for possible nonrandom distributions of antagonistic pairs and nonantagonistic pairs with respect to genome-wide recombination rates.

To test for significant differences in LD between antagonistic pairs and the two control pairs, we modelled variation in r^2^ as a declining exponential function of chromosomal distance and assessed differences in residual r^2^ (once distance was regressed out) using Wilcoxon rank-sum tests.

### Population genomic analyses for *D*. *simulans* and *D*. *yakuba*

We analysed polymorphism data from two closely related species from the *D*. *melanogaster* species group, *D*. *simulans* and *D*. *yakuba*, which are estimated to share a common ancestor with *D*. *melanogaster* approximately 1.5 million years and approximately 3 million years ago, respectively [55]. Our primary dataset for *D*. *simulans* consisted of 170 high-coverage whole-genome sequences from North American *D*. *simulans* inbred lines [56] (VCF files downloaded from https://zenodo.org/record/154261#.XEHMtM_7TUJ). These SNPs were further quality filtered (call rate >80%, depth >10, biallelic sites only) and allele frequencies estimated using vcftools [88]. The liftOver tool was then used to convert *D*. *simulans* to *D*. *melanogaster* coordinates.

A secondary dataset consisted of 20 high-coverage genomes from *D*. *simulans* and *D*. *yakuba* isofemale lines [57], respectively (downloaded from http://www.molpopgen.org/). These sequences are derived from flies sampled in each species’ African distribution range (Madagascar and Kenya in the case of *D*. *simulans*; Cameroon and Kenya in the case of *D*. *yakuba*). Whole-genome sequences from each individual were first aligned to the *D*. *melanogaster* Release 5 genome using Mauve [109]. We then filtered the alignments to keep only positions that were polymorphic and whose call rate was 100% across the 20 individuals of each species. We finally converted coordinates from *D*. *melanogaster* Release 5 to Release 6 using the liftOver tool.

To test whether antagonistic SNPs are associated with signatures of balancing selection in *D*. *simulans* and *D*. *yakuba*, we asked whether antagonistic sites were enriched among sites that were *trans*-specific. A *trans*-specific site was defined as a position where (i) polymorphism was detectable in LH_M_ and the species under consideration, and (ii) the allelic variants at that polymorphism matched those observed in LH_M_. If one or both of these conditions were not met, the site was categorised as ‘non-*trans*-specific’.

To compare the *trans*-specific status of antagonistic and nonantagonistic sites (Fig 6B, 6C and 6D), we replicated the Monte Carlo approach used to compare antagonistic/nonantagonistic MAFs among populations of *D*. *melanogaster* (i.e., analysis A in section ‘SNP-based analyses of balancing selection’)—that is, we sampled a set of LD-pruned nonantagonistic sites (LD clumping using r^2^ > 0.4, within 1 kb) and matched their (linked selection–corrected) MAFs in LH_M_ to those of antagonistic sites. For each of 1,000 sets of frequency-matched nonantagonistic sites, we recorded the proportion of sites that were *trans*-specific. The observed proportion of *trans*-specific antagonistic sites was then compared against this null distribution to generate an empirical *P* value.

To test whether *trans*-specific status varied with absolute GWAS effect size (Fig 6A), we mirrored analysis B described in ‘SNP-based analyses of balancing selection’. We performed a logistic regression with *trans*-specific status as the dependent variable and effect size the independent variable, with MAF in LH_M_ and linked selection included as covariates to account for ascertainment bias. We used a set of LD-pruned polymorphisms (LD clumping using r^2^ > 0.4, within 1 kb) for this analysis as well.

### Statistical software

All statistical analyses were carried out in RStudio (version 1.0.136 [110]). The analysis code is available at https://doi.org/10.5281/zenodo.2623225.

## Supporting information

S1 Fig Population structure and relatedness in LH_M_. (A) Scatterplot of the first and second principal components of a PCA constructed from SNPs present among LH_M_ (grey) and DGRP (red) populations. Principal components are computed from common (MAF > 0.05), LD-pruned (r^2^ > 0.2 within 10 kb) and high-quality (site-level call rate >95%) sites only. One notable outlier individual (black arrow) was removed prior to performing the GWAS. (B) Histogram of off-diagonal genomic relationship values between the 202 LH_M_ individuals retained for GWAS. Our sample consists of individuals with mostly low relatedness, with a small number of pairs of highly related individuals. (C) LD (measured as r^2^) in LH_M_ between pairs of SNPs situated within 1 kb of each other. Points represent mean r^2^ across 25-bp bins of distance; line represents a fitted declining exponential relationship between distance and r^2^. Data and code underlying this figure can be found at https://doi.org/10.5281/zenodo.2623225. DGRP, *Drosophila* Genetic Reference Panel; GWAS, genome-wide association study; LD, linkage disequilibrium; MAF, minor allele frequency; PCA, principal component analysis; SNP, single nucleotide polymorphism.
(TIF)

S2 Fig Testing for inflation due to population structure and relatedness. Q–Q plots of expected and observed *P* values from SNP-wise Wald *χ*^2^ tests for allelic effects on the antagonism index, based on a linear mixed model including the kinship matrix to correct for population structure and relatedness (purple dots), or on a simple linear model without kinship correction (black dots). The genomic inflation factor of the mixed model (*λ*_median_ = 0.967) indicates that population structure and relatedness have been well controlled for versus the simple linear model (*λ*_median_ = 1.209). Data and code underlying this figure can be found at https://doi.org/10.5281/zenodo.2623225. Q–Q, quantile-quantile; SNP, single nucleotide polymorphism.
(TIF)

S3 Fig Relationship between empirical and parametric *P* values. (A) SNP-wise *P* values obtained through a Wald *χ*^2^ test plotted against empirical *P* values obtained through 100,000 permutations of the kinship-scaled phenotypic values across individuals (see Materials and methods). The two sets of *P* values are very highly correlated (Pearson’s r > 0.999) and the regression coefficient (fitted line, pink) is very close to 1 (β = 0.996), indicating that parametric *P* values are robust. (B) Overlap between 2,372 candidate antagonistic SNPs—as defined from a Wald *χ*^2^ test and using a Q-value cutoff of 0.3—and the 2,372 sites with the lowest empirical *P* values. The near-perfect overlap between these two sets of sites (purple) reflects the strong positive correlation between parametric and empirical *P* values illustrated in A. and indicates that the candidate antagonistic SNPs defined through a parametric approach are robust. The mean Q-value across the 2,372 candidate antagonistic SNPs is comparable across both approaches, although somewhat higher when estimated from empirical *P* values (mean Q-value = 0.407) relative to parametric *P* values (mean Q-value = 0.267). Data and code underlying this figure can be found at https://doi.org/10.5281/zenodo.2623225. SNP, single nucleotide polymorphism.
(TIF)

S4 Fig Properties of variants mapping to the antagonism and concordant fitness indices. (A) Relative male and female lifetime reproductive fitness estimates for 223 *D*. *melanogaster* hemiclonal lines. Colours denote each line’s concordant index, i.e., their position along a spectrum (dashed arrow) ranging from male-detrimental, female-detrimental fitness effects (red) to male-beneficial, female-beneficial effects (blue). The concordant index is orthogonal to the antagonism index. (B) Histogram of Wald *χ*^2^ *P* values for variants mapped to the antagonism and concordant index. Variants associated with the antagonism index are significantly more enriched for very low *P* values than those associated with the concordant index. (C) Histogram of Q-values for the antagonism and concordant index. Vertical dashed line represents Q-value cutoff used for defining antagonistic/nonantagonistic sites. Data and code underlying this figure can be found at https://doi.org/10.5281/zenodo.2623225.
(TIF)

S5 Fig Permutation tests of median distance between antagonistic SNPs. Density curves depict the distribution of median distances between SNPs labelled 'antagonistic', across 1,000 permutations of labels. Permutation tests were performed separately for the autosomes and the X chromosome. Red lines show the observed median distance between antagonistic SNPs on the autosomes (147 bp) and the X chromosome (298 bp), respectively. Data and code underlying this figure can be found at https://doi.org/10.5281/zenodo.2623225. SNP, single nucleotide polymorphism.
(TIF)

S6 Fig Enrichment of antagonistic SNPs among chromosomal compartments and variant types. (A) Enrichment of antagonistic SNPs across individual chromosome arms. Results of Z-tests that compare the number of antagonistic candidate SNPs on each chromosome arm relative to all SNPs covered in the final SNP dataset are shown. (B) Same as A. but grouping autosomal chromosome arms together. (C) Enrichment of variant types among antagonistic SNPs within variant type categories (see http://www.ensembl.org/info/genome/variation/prediction/predicted_data.html for definitions). Shown are the results of Z-tests comparing the number of candidate antagonistic SNPs falling into each functional category against the representation of each category among all SNPs (see Materials and methods). For all plots, dark blue = statistically significant Z-test (*P* < 0.05), light blue = non-statistically significant Z-test (*P* > 0.05). Data and code underlying this figure can be found at https://doi.org/10.5281/zenodo.2623225. SNP, single nucleotide polymorphism.
(TIF)

S7 Fig SNP-based signatures of balancing selection in the ZI population, split by functional annotation. These analyses replicate Fig 4 but only consider SNPs situated in each functional category in turn. Excess polymorphism among antagonistic sites and positive relationships between excess polymorphism and GWAS effect size indicate that SNPs with effects on the antagonistic phenotype show elevated MAF. This can be the case even if a particular class of function is not more often (or even significantly less often) associated with antagonism than expected by chance (cf. Figs 2B and S6), indicating that the signal of balancing selection is due to the antagonistic effects of individual SNPs and not the general properties of its functional class. Data and code underlying this figure can be found at https://doi.org/10.5281/zenodo.2623225. GWAS, genome-wide association study; MAF, minor allele frequency; SNP, single nucleotide polymorphism; ZI, Zambia.
(TIF)

S1 Table List of antagonistic genes. Genes where antagonistic SNPs were found. This includes information on their genomic location, how many antagonistic/nonantagonistic SNPs fall in each gene, and whether the antagonistic/nonantagonistic SNPs have missense effects. SNP, single nucleotide polymorphism.
(XLSX)

## Abbreviations

DGRP: *Drosophila* Genetic Reference Panel
FDR: false discovery rate
GLM: generalised linear model
GO: Gene Ontology
GWAS: genome-wide association study
LD: linkage disequilibrium
LRT: likelihood ratio test
MAF: minor allele frequency
PCA: principal component analysis
PPI: protein–protein interaction
REML: restricted maximum likelihood
SA: South Africa
SNP: single nucleotide polymorphism
UTR: untranslated region
ZI: Zambia
