# Supplementary figures and images for: Genome-wide sexually antagonistic variants reveal long-standing constraints on sexual dimorphism in fruit flies
Source: PLoS Biol. 2019 Apr 25;17(4):e3000244. doi: 10.1371/journal.pbio.3000244 (PMC6504117; doi:10.1371/journal.pbio.3000244)

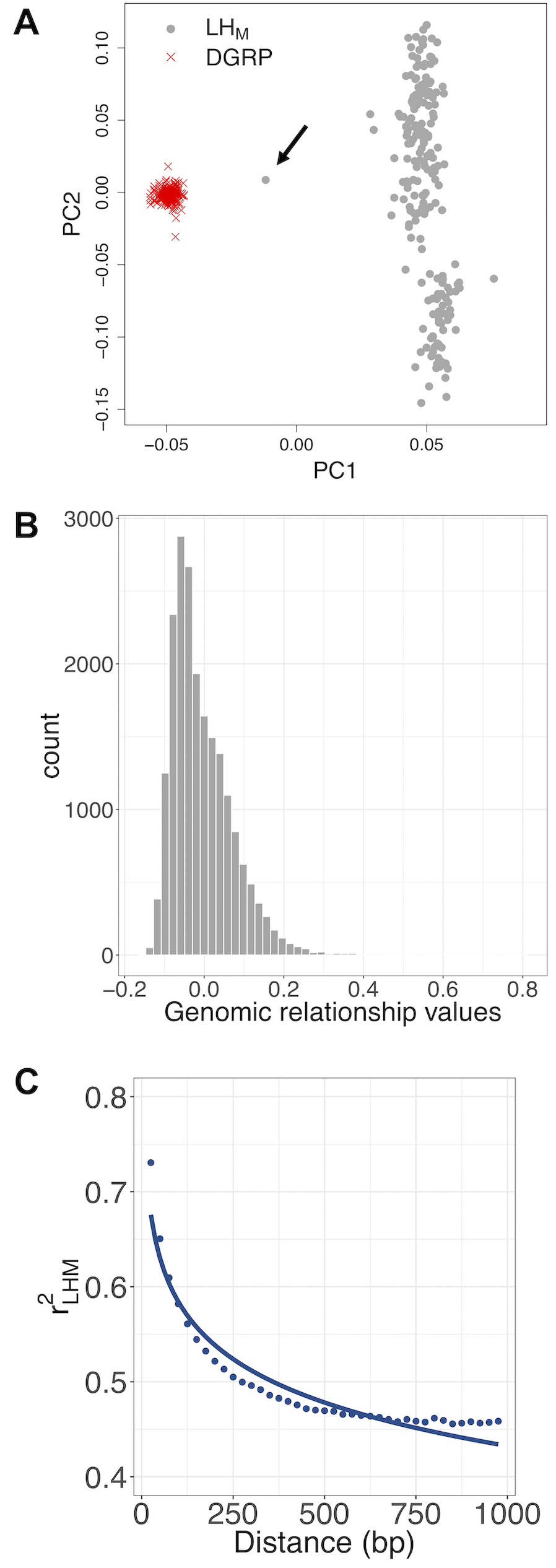

Supplement: S1 Fig — (A) Scatterplot of the first and second principal components of a PCA constructed from SNPs present among LHM (grey) and DGRP (red) populations. Principal components are computed from common (MAF > 0.05), LD-pruned (r2 > 0.2 within 10 kb) and high-quality (site-level call rate >95%) sites only. One notable outlier individual (black arrow) was removed prior to performing the GWAS. (B) Histogram of off-diagonal genomic relationship values between the 202 LHM individuals retained for GWAS. Our sample consists of individuals with mostly low relatedness, with a small number of pairs of highly related individuals. (C) LD (measured as r2) in LHM between pairs of SNPs situated within 1 kb of each other. Points represent mean r2 across 25-bp bins of distance; line represents a fitted declining exponential relationship between distance and r2. Data and code underlying this figure can be found at https://doi.org/10.5281/zenodo.2623225. DGRP, Drosophila Genetic Reference Panel; GWAS, genome-wide association study; LD, linkage disequilibrium; MAF, minor allele frequency; PCA, principal component analysis; SNP, single nucleotide polymorphism. (TIF) [file pbio.3000244.s001.tif]

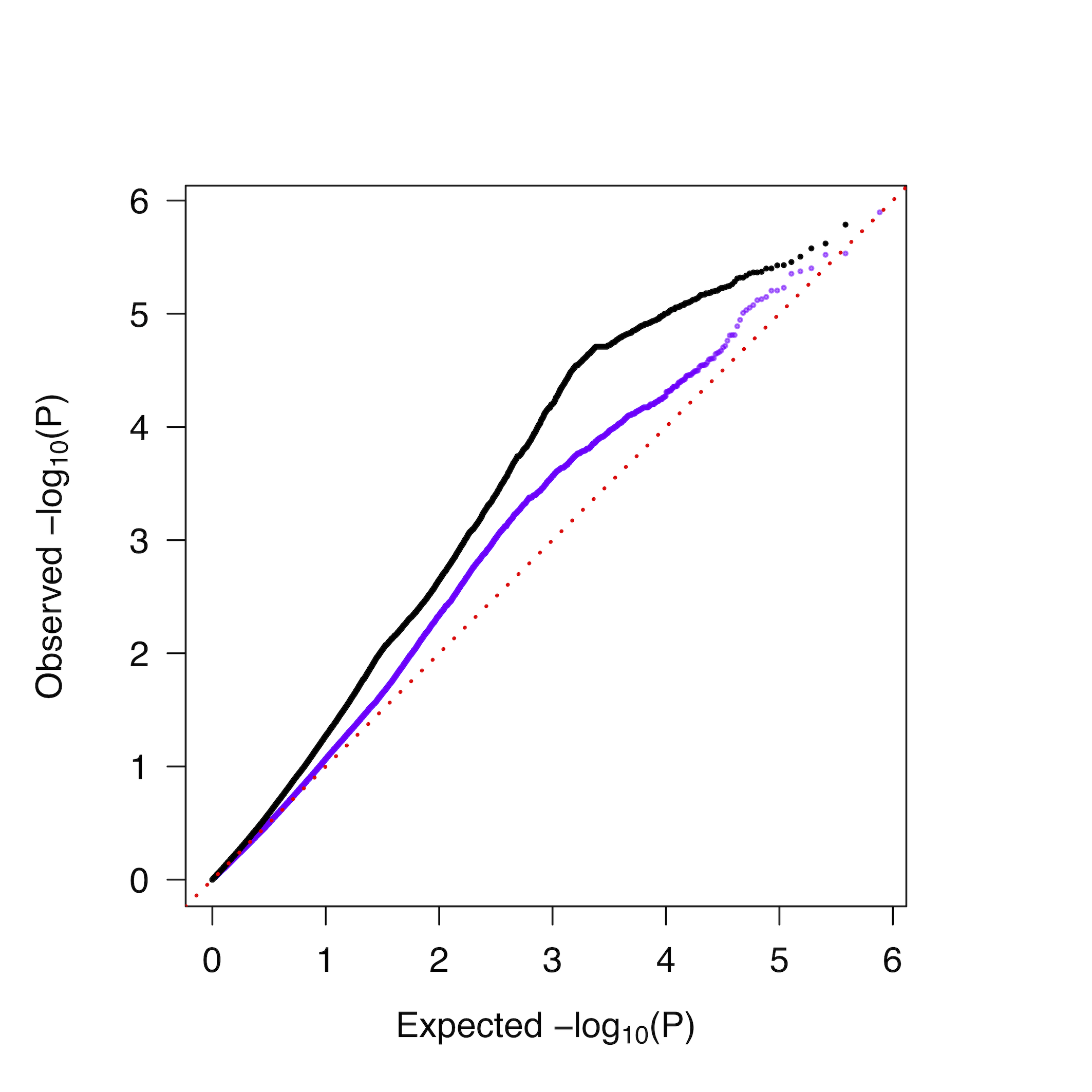

Supplement: S2 Fig — Q–Q plots of expected and observed P values from SNP-wise Wald χ2 tests for allelic effects on the antagonism index, based on a linear mixed model including the kinship matrix to correct for population structure and relatedness (purple dots), or on a simple linear model without kinship correction (black dots). The genomic inflation factor of the mixed model (λmedian = 0.967) indicates that population structure and relatedness have been well controlled for versus the simple linear model (λmedian = 1.209). Data and code underlying this figure can be found at https://doi.org/10.5281/zenodo.2623225. Q–Q, quantile-quantile; SNP, single nucleotide polymorphism. (TIF) [file pbio.3000244.s002.tif]

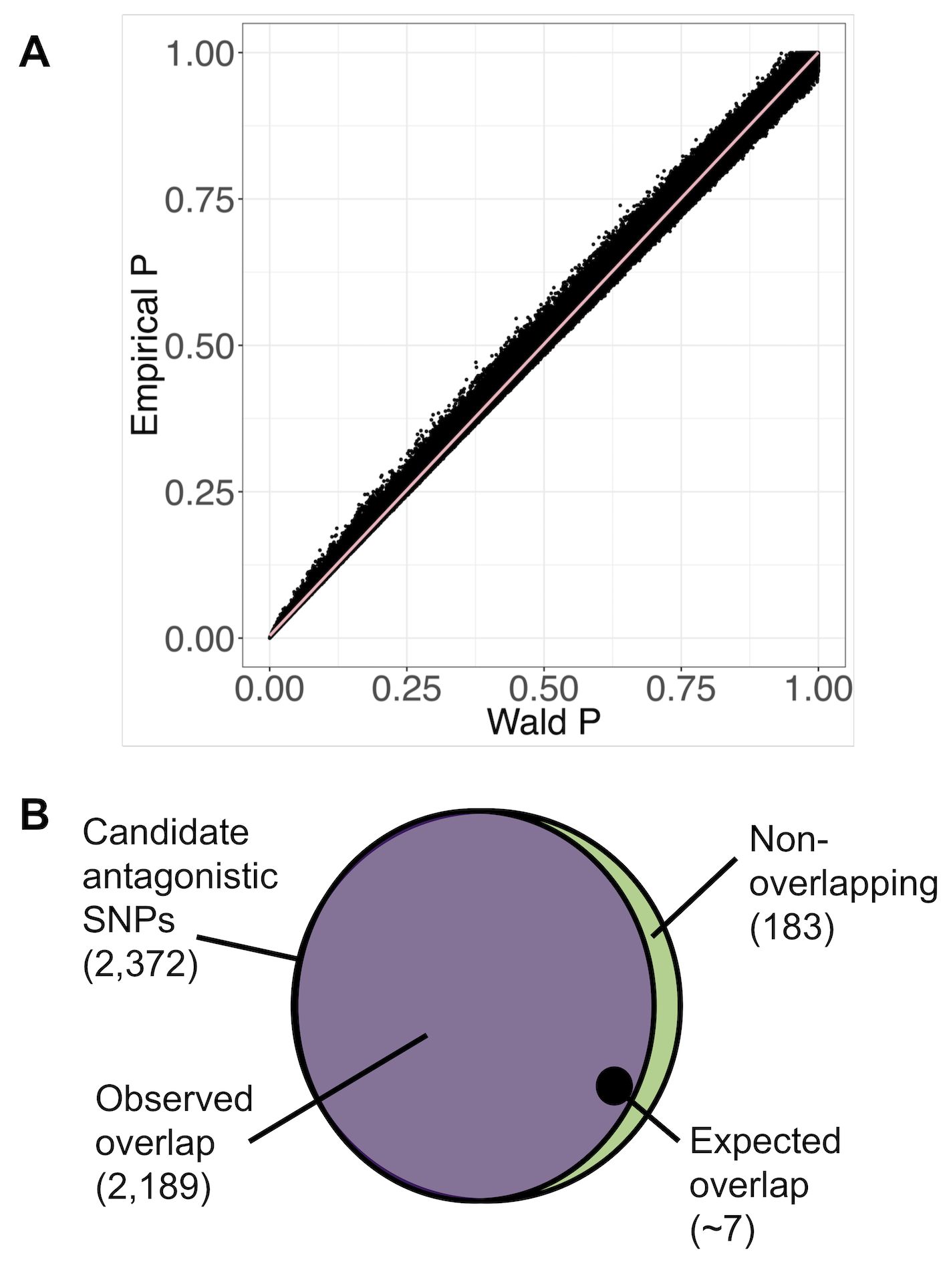

Supplement: S3 Fig — (A) SNP-wise P values obtained through a Wald χ2 test plotted against empirical P values obtained through 100,000 permutations of the kinship-scaled phenotypic values across individuals (see Materials and methods). The two sets of P values are very highly correlated (Pearson’s r > 0.999) and the regression coefficient (fitted line, pink) is very close to 1 (β = 0.996), indicating that parametric P values are robust. (B) Overlap between 2,372 candidate antagonistic SNPs—as defined from a Wald χ2 test and using a Q-value cutoff of 0.3—and the 2,372 sites with the lowest empirical P values. The near-perfect overlap between these two sets of sites (purple) reflects the strong positive correlation between parametric and empirical P values illustrated in A. and indicates that the candidate antagonistic SNPs defined through a parametric approach are robust. The mean Q-value across the 2,372 candidate antagonistic SNPs is comparable across both approaches, although somewhat higher when estimated from empirical P values (mean Q-value = 0.407) relative to parametric P values (mean Q-value = 0.267). Data and code underlying this figure can be found at https://doi.org/10.5281/zenodo.2623225. SNP, single nucleotide polymorphism. (TIF) [file pbio.3000244.s003.tif]

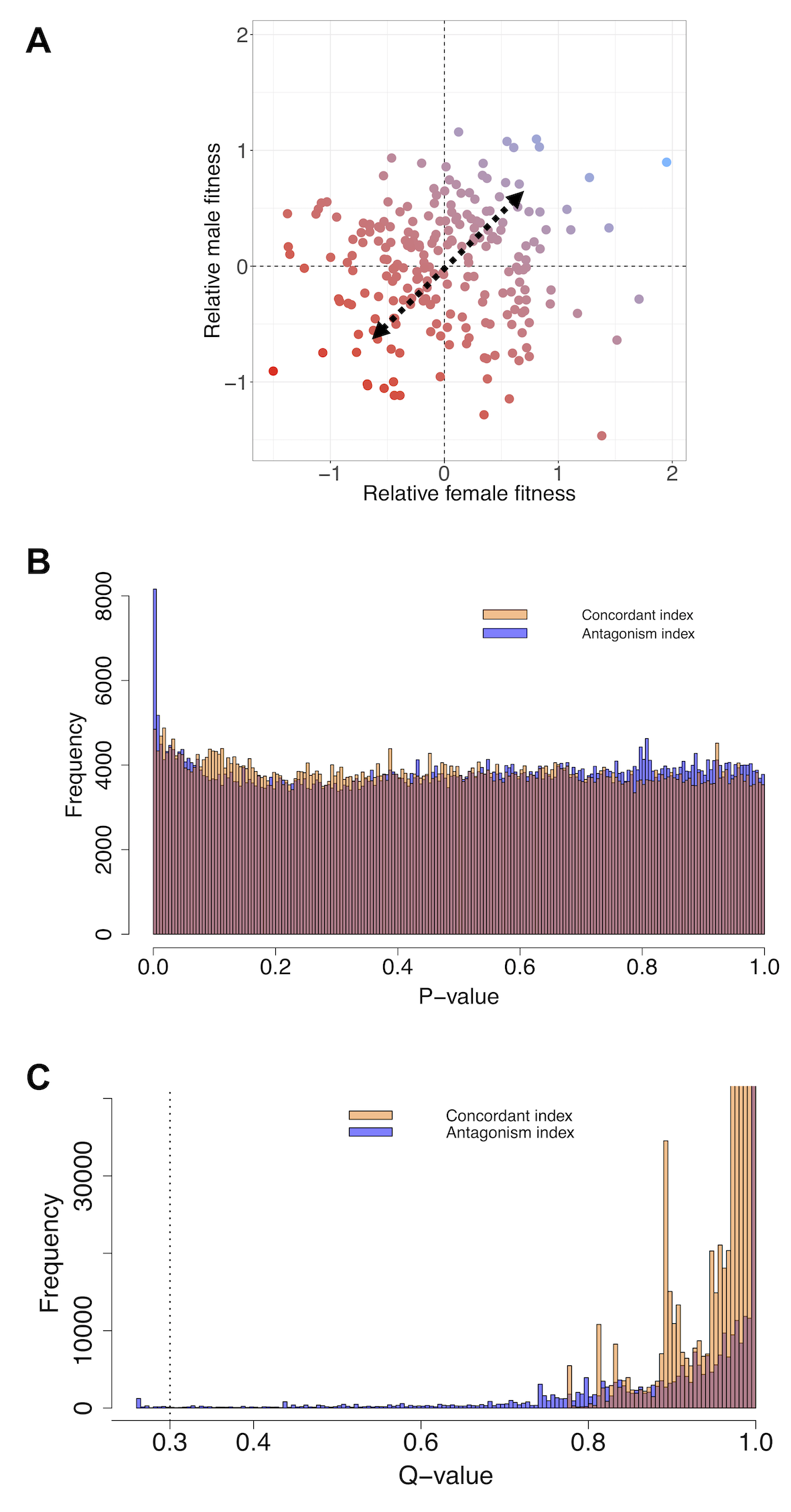

Supplement: S4 Fig — (A) Relative male and female lifetime reproductive fitness estimates for 223 D. melanogaster hemiclonal lines. Colours denote each line’s concordant index, i.e., their position along a spectrum (dashed arrow) ranging from male-detrimental, female-detrimental fitness effects (red) to male-beneficial, female-beneficial effects (blue). The concordant index is orthogonal to the antagonism index. (B) Histogram of Wald χ2 P values for variants mapped to the antagonism and concordant index. Variants associated with the antagonism index are significantly more enriched for very low P values than those associated with the concordant index. (C) Histogram of Q-values for the antagonism and concordant index. Vertical dashed line represents Q-value cutoff used for defining antagonistic/nonantagonistic sites. Data and code underlying this figure can be found at https://doi.org/10.5281/zenodo.2623225. (TIF) [file pbio.3000244.s004.tif]

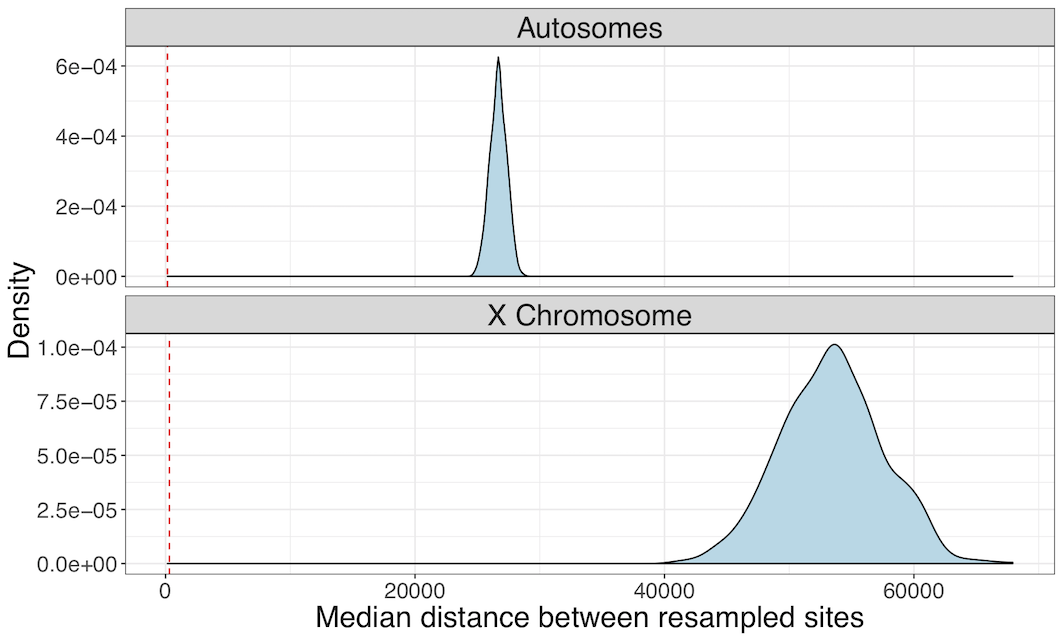

Supplement: S5 Fig — Density curves depict the distribution of median distances between SNPs labelled 'antagonistic', across 1,000 permutations of labels. Permutation tests were performed separately for the autosomes and the X chromosome. Red lines show the observed median distance between antagonistic SNPs on the autosomes (147 bp) and the X chromosome (298 bp), respectively. Data and code underlying this figure can be found at https://doi.org/10.5281/zenodo.2623225. SNP, single nucleotide polymorphism. (TIF) [file pbio.3000244.s005.tif]

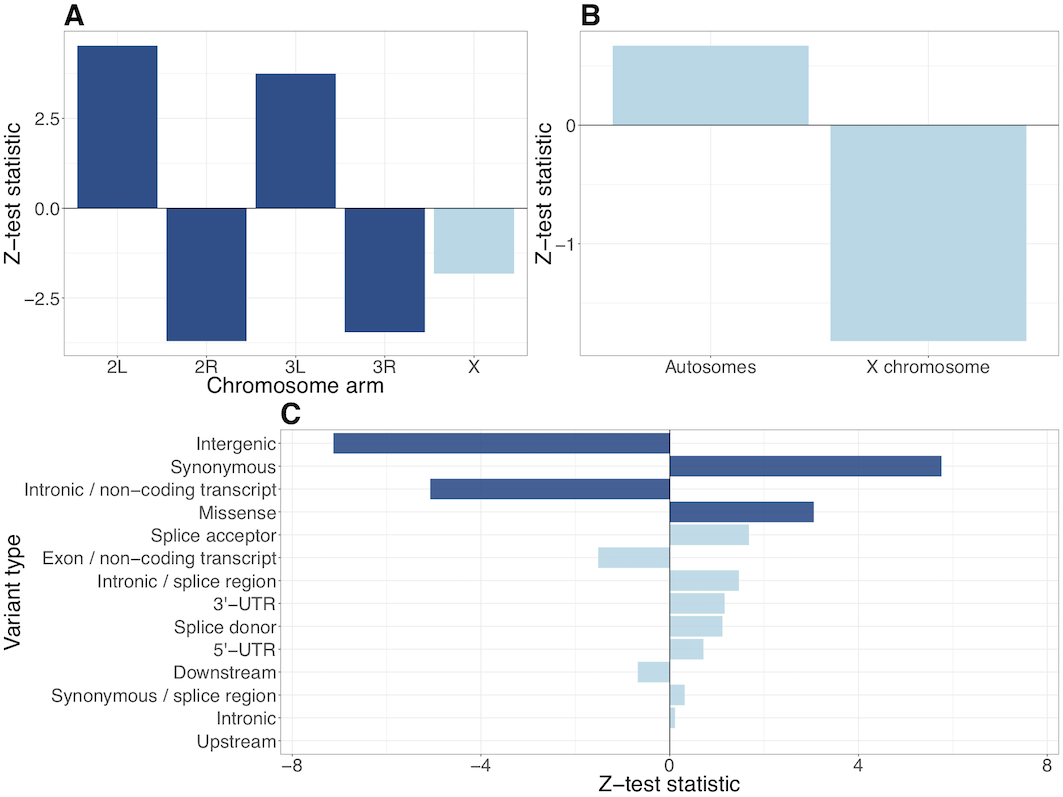

Supplement: S6 Fig — (A) Enrichment of antagonistic SNPs across individual chromosome arms. Results of Z-tests that compare the number of antagonistic candidate SNPs on each chromosome arm relative to all SNPs covered in the final SNP dataset are shown. (B) Same as A. but grouping autosomal chromosome arms together. (C) Enrichment of variant types among antagonistic SNPs within variant type categories (see http://www.ensembl.org/info/genome/variation/prediction/predicted_data.html for definitions). Shown are the results of Z-tests comparing the number of candidate antagonistic SNPs falling into each functional category against the representation of each category among all SNPs (see Materials and methods). For all plots, dark blue = statistically significant Z-test (P < 0.05), light blue = non-statistically significant Z-test (P > 0.05). Data and code underlying this figure can be found at https://doi.org/10.5281/zenodo.2623225. SNP, single nucleotide polymorphism. (TIF) [file pbio.3000244.s006.tif]

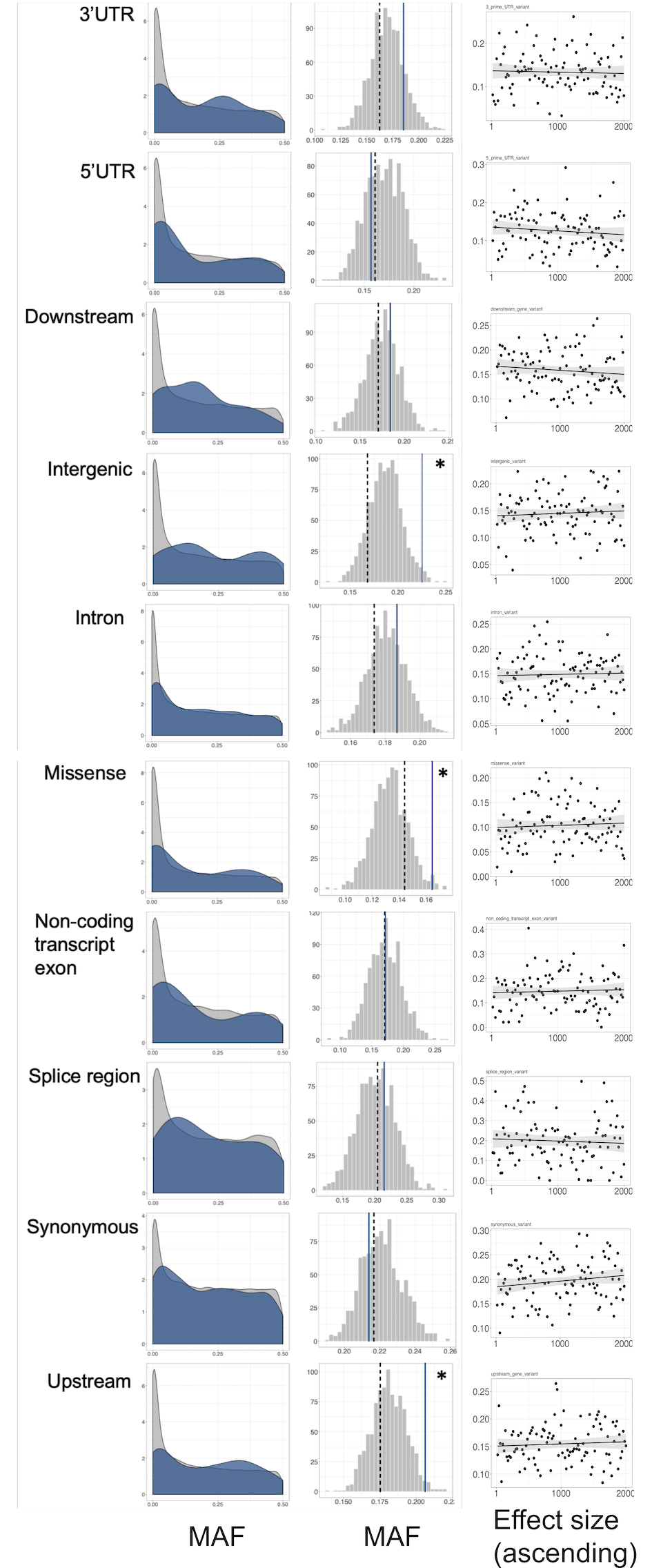

Supplement: S7 Fig — These analyses replicate Fig 4 but only consider SNPs situated in each functional category in turn. Excess polymorphism among antagonistic sites and positive relationships between excess polymorphism and GWAS effect size indicate that SNPs with effects on the antagonistic phenotype show elevated MAF. This can be the case even if a particular class of function is not more often (or even significantly less often) associated with antagonism than expected by chance (cf. Figs 2B and S6), indicating that the signal of balancing selection is due to the antagonistic effects of individual SNPs and not the general properties of its functional class. Data and code underlying this figure can be found at https://doi.org/10.5281/zenodo.2623225. GWAS, genome-wide association study; MAF, minor allele frequency; SNP, single nucleotide polymorphism; ZI, Zambia. (TIF) [file pbio.3000244.s007.tif]
